# Association of GC Variants with Bone Mineral Density and Serum VDBP Concentrations in Mexican Population

**DOI:** 10.3390/genes12081176

**Published:** 2021-07-29

**Authors:** Berenice Rivera-Paredez, Alberto Hidalgo-Bravo, Guadalupe León-Reyes, Bárbara Antuna-Puente, Yvonne N. Flores, Jorge Salmerón, Rafael Velázquez-Cruz

**Affiliations:** 1 Research Center in Policies, Population and Health, School of Medicine, National Autonomous University of Mexico (UNAM), Mexico 04510, Mexico; bereriveraparedez7@gmail.com (B.R.-P.); jorge.salmec@gmail.com (J.S.); 2 Department of Genetics, National Institute of Rehabilitation (INR), Mexico 014389, Mexico; dr_genetica@yahoo.com; 3 Genomics of Bone Metabolism Laboratory, National Institute of Genomic Medicine (INMEGEN), Mexico 14610, Mexico; greyes@inmegen.gob.mx; 4 Laboratory of Genomics of Cardiovascular Diseases, National Institute of Genomic Medicine (INMEGEN), Mexico 14610, Mexico; bantuna@inmegen.gob.mx; 5 Epidemiological and Health Services Research Unit, Morelos Delegation, Mexican Institute of Social Security, Cuernavaca, Morelos 62000, Mexico; ynflores@ucla.edu; 6 University of California, Los Angeles (UCLA), Department of Health Policy and Management and UCLA-Kaiser Permanente Center for Health Equity, Fielding School of Public Health, Los Angeles, CA 90095, USA; 7 UCLA Center for Cancer Prevention and Control Research, Fielding School of Public Health and Jonsson Comprehensive Cancer Center, Los Angeles, CA 90095, USA

**Keywords:** *GC* gene, vitamin D binding protein, bone mineral density, Mexican population, sex

## Abstract

Vitamin D-binding protein (VDBP) is encoded by the *GC* gene and is an active participant in the control of bone metabolism. However, the effect of its major variants on VDBP concentration and bone mineral density (BMD) remains unclear. Our aim was to analyze the effect of major *GC* variants on serum VDBP concentration and BMD. We recruited individuals from the Health Workers Cohort Study, which includes employees of the Mexican Institute of Social Security (IMSS). A total of 1853 adults were included. The single nucleotide polymorphisms (SNPs) rs7041 and rs4588 were genotyped to identify the three best characterized haplotypes of *GC*. Serum VBDP, 25(OH)D and BMD were also measured. Among women, the G allele of rs7041 was associated with higher VDBP and BMD compared to homozygous TT. The A allele of rs4588 was associated with lower VDBP and BMD compared to CC homozygous. In men, *GC* variants were only associated with VDBP levels. We did not observe an association between free/bioavailable 25(OH)D and BMD in men and women. Our results support an association of VDBP in bone health. The G and C alleles, from rs7041 and rs4588, respectively, are associated with high concentrations of VDBP and BMD in this sample of Mexican postmenopausal women.

## 1. Introduction

Vitamin D Binding Protein (VDBP) is the main transporter for Vitamin D (VitD) metabolites in serum; VDBP is also known as GC globulin and it is encoded by the *GC* gene localized in the locus 4q11-q13 [1]. The *GC* gene is known for being highly polymorphic with more than 120 different allelic variants identified [2]. Among these variants, three well-known haplotypes represent the most common and best characterized forms, which result from the combination of two single nucleotide polymorphisms (SNPs), rs7041 and rs4588. The SNP rs7401 is a T/G transversion resulting in the substitution of an asparagine for glutamine at position 432. The rs4588 SNP is a C/A transversion resulting in the change of a threonine for lysine at position 436. The combination of these two SNPs results in the following haplotypes and amino acids composition, GC1F (rs7041-T/rs4588-C; Asp/Lys); GC1S (rs7041-G/rs4588-C; Glu/Thr); and GC2 (rs7041-T/rs4588-A; Asp/Lys) [3]. The frequency of these three major haplotypes varies between populations. Despite the variation, the GC2 (rs7041-T/rs4588-A) haplotype tends to be the less frequent across populations, while GC1F (rs7041-T/rs4588-C) is the most common in Africa and GC1S (rs7041-G/rs4588-C) in Europe [4]. The biological significance of the three main *GC* haplotypes on its expression and function has been extensively investigated. Previous reports have found the highest VDBP serum concentration in carriers of the GC1 haplotype (either 1F (rs7041-T/rs4588-C) or 1S (rs7041-G/rs4588-C)), and the lowest in homozygous for the GC2 (rs7041-T/rs4588-A) haplotype [5].

Furthermore, the serum concentration of VitD metabolites also varies among carriers of the different *GC* haplotypes. The concentration of 1α, 25-dihydroxyvitamin D (1,25(OH)2D) and 25-hydroxyvitamin D (25(OH)D) tends to be higher in GC1-1 carriers compared to homozygous GC2-2; this finding was observed only with the monoclonal method in an African population [6]. Considering the effect of the GC1 and GC2 haplotypes on the serum concentrations of VDBP and VitD, numerous studies have been conducted to investigate the functional consequences of the *GC* haplotypes. The role of VitD and its carrier on Bone Mineral Density (BMD) has been a major focus of various studies. Osteoporosis is defined as a BMD T-score of −2.5 or less [7]. The characteristic of osteoporosis is microarchitecture deterioration and bone fragility [8]. Current evidence suggests that BMD is influenced by VitD and serum VDBP concentration [9,10]. However, the contribution of the *GC* variants and 25(OH)D on BMD remains controversial. Elucidation of this relationship is critical to gain a better understanding of the factors predisposing to BMD loss and subsequent fragility fractures.

As part of a proteomic analysis previously carried out by our group, we identified VDBP as a potential biomarker for low BMD in Mexican postmenopausal women [11]. Additionally, different studies have shown that women have higher VDBP serum concentrations than men [12]. The aim of the present study was to investigate the effect of the major *GC* variants on serum concentration of VDBP and BMD in a cohort of Mexican Mestizo individuals. Our results suggest an association of the two main *GC* variants with VDBP serum concentration and BMD in Mexican Mestizo women.

## 2. Materials and Methods

### 2.1. Study Population

The Health Workers Cohort Study (HWCS) is a prospective cohort study investigating the association between genetic and lifestyle factors with the development of chronic diseases in Mexican Mestizo individuals. Details of this cohort have been described previously [13]. Briefly, this study was conducted at the Mexican Institute of Social Security (IMSS) in Morelos, México, with three data collection stages: 2004–2006, 2010–2016 and 2017–2018. This cohort study was approved by the ethics committee of the IMSS and informed consent was obtained from all participants. The study population for the present analysis consists of adults who participated in the second stage of the HWSC and provided a DNA sample. Exclusion criteria were: less than 18 years old (*n* = 85), missing data of VDBP levels (*n* = 28) or BMD (*n* = 119). A total of 1853 participants were included in the analysis.

### 2.2. Biochemical Analysis

Blood was drawn after 8 h of fasting. Serum VDBP was measured by ELISA, using a commercial kit (Quantikine ELISA kit (R&D Systems, Minneapolis, MN, USA, Cat No. DVDBP0B) (intra- and inter-assay coefficient of variation, <7%) [11]. A recent recommendation by the International Federation of Clinical Chemistry has established that immunoassay is a reliable method for VDBP quantification not biased for genotypes [14].

Serum 25(OH)D was measured with LIAISON^®^ 25OH Vitamin D Total Assay (Diasorin, Saluggia (VC), Italy) (intra- and inter-assay variation coefficients < 10%) [15]. Albumin was measured by a colorimetric method (bromocresol green) using a UniCel^®^ DxC 600/800 System(s) and Synchron^®^ Systems Multi Calibrator, Beckman Coulter (intra- and inter-assay coefficient of variation, <4.5%) [16]. VitD status was defined as normal with serum levels of 25(OH)D ≥ 50 nmol/L and VitD deficiency < 50.0 nmol/L (20.0 ng/mL) [17]. Free (pg/mL) and bioavailable 25(OH)D (ng/mL) were calculated by VDBP and albumin determinations, using a formula adjusted for the *GC* gene haplotypes [18,19]. The equations used are:

$${\mathrm{Free}}_{25\left(\mathrm{OH}\right)D}=\frac{\mathrm{Total}\;25\left(\mathrm{OH}\right)D}{1+\left(6\times 10^{5}M^{-1}\right)\times \mathrm{albumin}\times \left(\mathrm{Affinity}\;\mathrm{GC}\;\mathrm{gene}\right)\times \mathrm{VDBP}}$$

$${\mathrm{Bioavailable}}_{25\left(\mathrm{OH}\right)D}=\left[\left(\left(6\times 10^{5}M^{-1}\right)\times \mathrm{albumin}\right)+1\right]\times {\mathrm{Free}}_{25\left(\mathrm{OH}\right)D}$$

We used the following affinity coefficients for *GC* gene haplotypes, previously reported as: 1S/1S = 6 × 10^8^, 1S/1F = 4.8 × 10^8^, 1S/2 = 8.6 × 10^8^, 1F/1F = 3.6 × 10^8^, 1F/2 = 7.4 × 10^8^, 2/2 = 11.2 × 10^8^ [18].

### 2.3. BMD Measurements

Participants underwent dual-energy X-ray absorptiometry (DXA; Lunar DPX-GE, Lunar Radiation, software version 1.35, fast scan mode) to determine femoral neck, hip and lumbar spine BMD in g/cm^2^ and their T-scores were calculated [13]. The procedures were performed according to the manufacturer’s instructions by experienced technicians, who ensured that the daily variation coefficient was within normal operational standards and in vivo variation coefficient was lower than 1.5%. Low BMD was defined as a T-score less than −1 at the femoral neck, lumbar spine and hip, according to WHO criteria [7].

### 2.4. Genotyping of GC Gene Polymorphisms

DNA was extracted from peripheral blood using the QIAamp DNA Blood Mini Kit according to manufacturer’s instructions. Two *GC* gene variants, rs4588 and rs7041, were genotyped. Genotyping was carried out using predesigned TaqMan SNP Genotyping assays (Applied Biosystems, Massachusetts, MA, USA), in a QuantStudio 7 Flex Real-Time PCR system (Applied Biosystems, Massachusetts, MA, USA). Automatic variant call was carried out by the SDS software version 2.2.1.

### 2.5. Other Measurements

Demographic, clinical and lifestyle data were obtained through self-administered questionnaires [13]. Height was measured using a standing stadiometer (SECA). Weight was measured using a calibrated scale (model BC-533; Tanita, IL, USA). Body mass index (BMI) was calculated as weight (kg/height (m)^2^). VitD intake during the past year was assessed with a previously validated 116-item semiquantitative food frequency questionnaire [20,21].

### 2.6. Statistical Analysis

Descriptive data were expressed as mean (standard deviation), median (interquartile range) or percentage for continuous or categorical variables, as appropriate. Adjusted medians (95% confidence interval) were derived from multivariable quantile regression models that included the following variables: age groups, sex, body mass index, leisure time physical activity, smoking status and VitD intake.

To explore the determinants of VDBP levels, we used univariable and multivariable quantile regressions, modeling VDBP as a continuous variable. This nonparametric statistical method models the median of the outcome variable and any other percentile across the distribution without the need to categorize the variable. The factors evaluated were age, physical activity, smoking status, BMI categories, VitD intake, VitD levels, alcohol intake and the rs4588 and rs7041 genetic variants.

We estimated the distribution of VDBP levels and BMD, at different body regions, in the rs4588 and rs7041 variants. Additionally, logistic regression analysis and linear regression adjusted for relevant confounders were performed to evaluate the association between genetic variants of the *GC* gene and BMD.

Spearman’s rho correlation coefficients were calculated between VDBP levels, the forms of 25(OH)D and BMD at different body regions. To determine the association between VDBP categories defined by tertiles and low BMD, we used logistic regression analysis. These models were adjusted by age groups, sex, BMI, leisure time physical activity, smoking status, alcohol intake, VitD intake and 25(OH)D levels.

Non-normally distributed variables such as VDBP and 25(OH)D metabolites (total, free, and bioavailable) were logarithmically transformed to base 10 to meet the assumptions of parametric statistical tests prior to data analysis. We evaluated the association between VDBP levels, different forms of VitD and BMD at different body regions using a linear regression. The VDBP and BMD models were adjusted for age groups, sex, BMI, alcohol, leisure time physical activity, smoking status, VitD intake and 25(OH)D levels; the models with different forms of VitD and BMD were adjusted by age groups, sex, BMI categories, alcohol, leisure time physical activity, smoking status and VitD intake.

We evaluated the interaction between nutrient categories (VitD, calcium, magnesium, potassium, phosphorous, protein intake) and VDBP categories defined by tertiles and low BMD. This analysis was performed by introducing the interaction term as a covariate in the logistic regression models. The testparm command tested (Wald test) the significance of the interaction.

All statistical analyses were performed on all participants, stratifying by sex, postmenopausal status, and VitD status. Stata software was used for data analyses. Graphics were performed with GraphPad Prism (version 6). A *p* value < 0.05 was considered statistically significant.

## 3. Results

### 3.1. Description of the Sample

This study included 1853 participants from the HWCS, of which 69.6% were women and 30.4% were men. The median of VDBP levels was higher among women than men, while the median of total, free and bioavailable 25(OH)D was lower in women than men (Table S1). Demographic characteristics are presented by sex and VDBP tertiles (Table 1). Women in the lowest VDBP category, defined by tertiles, were older and had a higher prevalence of low BMD at the femoral neck and hip compared to women in the highest VDBP category.

On the other hand, no significant differences were observed for these measurements among men. The adjusted median of free and bioavailable 25(OH)D was lower in women and men, who were in the highest VDBP category. The minor allele frequency (MAF) of rs4588 was also lower in women and men in the highest VDBP category, while the MAF of rs7041 was higher only in women within the highest VDBP category (*p* < 0.05). Additionally, we found significant differences in VDBP levels between women and men after stratifying by age groups.

### 3.2. Determinants of VDBP Levels

We observed that age and genetic variants of the *GC* gene were consistently associated with VDBP levels in all evaluated groups. However, we did not observe consistency for associations with physical activity, smoking status, BMI categories, VitD levels, VitD intake and alcohol (Tables S2–S8).

**Figure 1:**
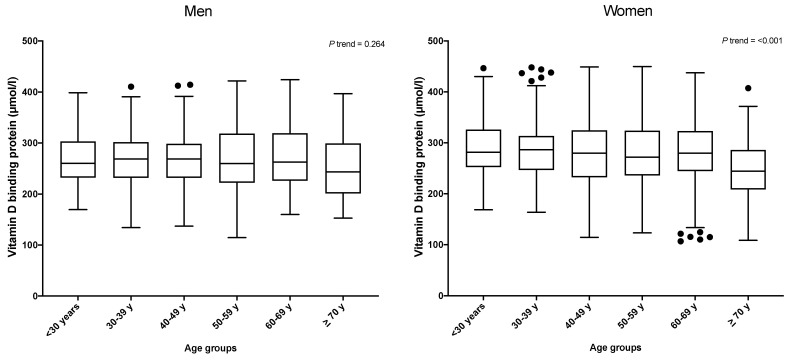
VDBP levels by age groups and sex. Black dots are extreme values from each category.

Among women, the VDBP concentration was found to decrease dramatically with age (*p* trend < 0.001), but this trend was not observed in men (*p* trend = 0.264) (Figure 1).

**Figure 2:**
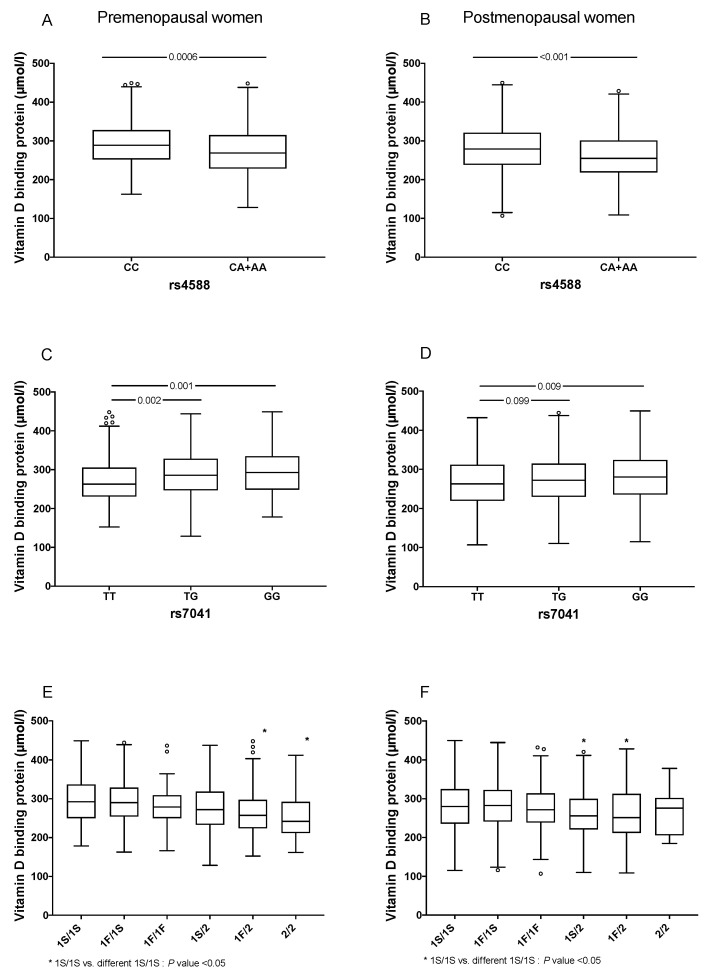
Comparison of VDBP levels by GC gene variants and menopausal status. (**A**) VDBP levels in premenopausal women carrying of rs4588 locus genotypes, (**B**) VDBP levels in post-menopausal women carrying of rs4588 locus genotypes, (**C**) VDBP levels in premenopausal women carrying of rs7041 locus genotypes, (**D**) VDBP levels in postmenopausal women carrying of rs7041 locus genotypes, (**E**) VDBP levels in premenopausal women carrying of GC haplotypes and (**F**) VDBP levels in postmenopausal women carrying of GC haplotypes. Traditional nomen-clature referring to the protein phenotypes: (a) GC1F (rs7041-T/rs4588-C); (b) GC1S (rs7041-G/rs4588-C) and (c) GC2 (rs7041-T/rs4588-A). The symbol ° represent extreme values from each category.

Individuals carrying at least one A allele in rs4588, had lower levels of VDBP than CC individuals (249.0 vs. 270.2 μmol/L in men and 260.0 vs. 282.0 μmol/l in women, respectively) (Figure S1A,B). Additionally, carriers of the GG genotype of rs7041 had significant higher levels of VDBP when compared to the TT carriers (272.7 vs. 247.3 μmol/L in men and 284.1 vs. 262.6 μmol/L in women, respectively, *p* < 0.001) (Figure S1C,D). We then analyzed the association of VDBP concentrations considering the haplotypes determined by rs4588 and rs7041. In women, carriers of the 1S/2, 1F/2 and 2/2 haplotypes had lower medians of VDBP concentrations compared to the 1S/1S haplotype, while among men, only the 1F/2 haplotype showed a statistically significant difference (Figure S1E,F). When stratified by menopausal status, directionality of our results remained the same (Figure 2).

### 3.3. Association between GC Gene and BMD

**Figure 3:**
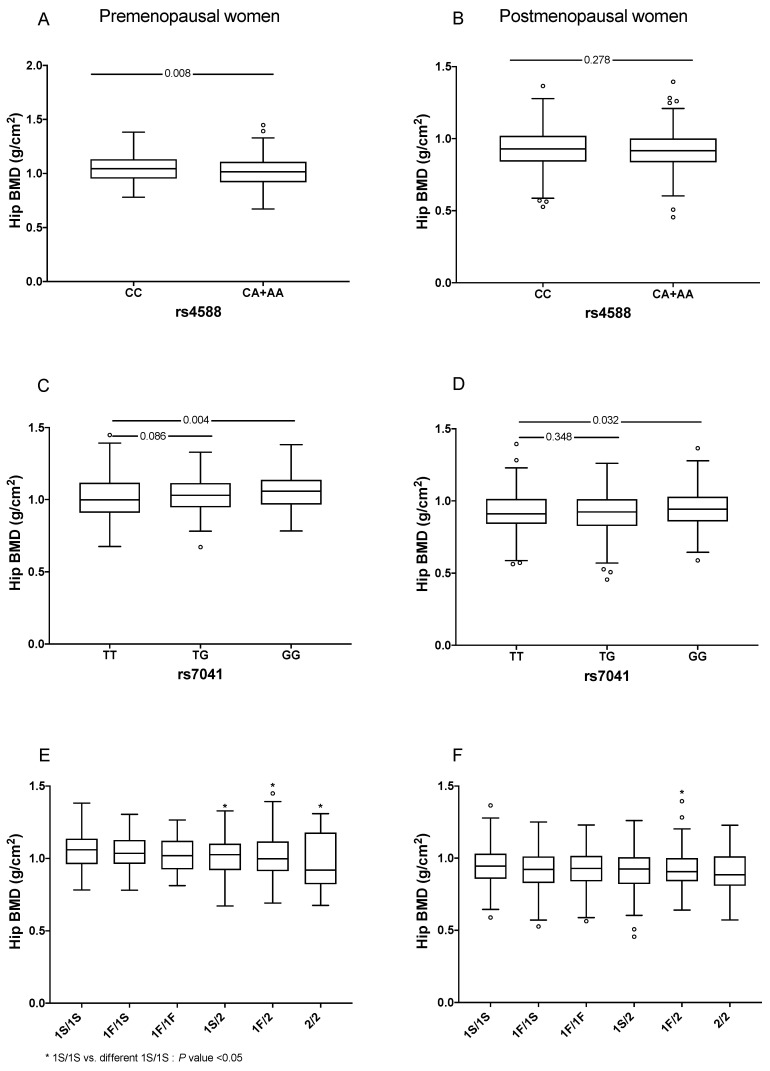
Comparison of hip BMD by GC gene variants by menopausal status. (**A**) Hip BMD in premenopausal women carrying of rs4588 locus genotypes, (**B**) Hip BMD in postmenopausal women carrying of rs4588 locus genotypes, (**C**) Hip BMD in premenopausal women carrying of rs7041 locus genotypes, (**D**) Hip BMD in postmenopausal women carrying of rs7041 locus geno-types, (**E**) Hip BMD in premenopausal women carrying of GC haplotypes and (**F**) Hip BMD in postmenopausal women carrying of GC haplotypes. Traditional nomenclature referring to the protein phenotypes: (a) GC1F (rs7041-T/rs4588-C); (b) GC1S (rs7041-G/rs4588-C) and (c) GC2 (rs7041-T/rs4588-A). The symbol ° represent extreme values from each category.

We explored the distribution of BMD by *GC* genetic variants and haplotypes without adjustment for covariates. Premenopausal women carrying at least one A allele of rs4588 had significant lower levels of BMD at the hip (1.011 vs. 1.045, *p* = 0.008) and femoral neck (1.018 vs. 0.999, *p* = 0.004) compared to the CC carriers. However, this difference was not statistically significant in postmenopausal women (0.917 vs. 0.929, *p* = 0.278 for hip BMD and 0.875 vs. 0.869, *p* = 0.228 for femoral neck BMD). In contrast, carriers of the GG genotype of SNP rs7041 had higher BMD at the hip compared to the TT carriers (1.058 vs. 0.998, *p* = 0.004 in premenopausal women and 0.942 vs. 0.910, *p* = 0.032 in postmenopausal women) (Figure 3). In contrast, the carriers of the GG genotype of SNP rs7041 had higher levels of femoral neck BMD compared to the TT carriers (0.991 vs. 1.029, *p* = 0.018, in premenopausal women and 0.868 vs. 0.898, *p* = 0.015, among postmenopausal women). We also analyzed hip BMD by haplotypes and found that premenopausal women carrying at least one copy of the GC2 (rs7041-T/rs4588-A) haplotype had significant differences in BMD, compared to homozygous 1S/1S. Among postmenopausal women, with at least one copy of the GC2 (rs7041-T/rs4588-A) haplotype significantly lowered hip BMD, compared to the homozygous 1S/1S (Figure 3). Similar results were observed for the femoral neck but not for the lumbar spine (Figures S2 and S3).

We also investigated the association between genotypes and BMD as a categorical or continuous variable adjusting for covariates (Tables S9–S11). In all participants, we found a risk association between the GC 2/2 genotype and low BMD at the hip in the crude and adjusted models (OR = 2.75, 95% CI: 1.55–5.00 and OR= 2.40, 95% CI: 1.19–4.80, respectively) (Table S9). In all women, under the codominant model, we observed a negative association between the GG genotype of rs7041 and low BMD at the hip (OR = 0.62, 95% CI: 0.41–0.94, *p* = 0.025). Additionally, we found a risk association between the GC 2/2 genotype and low BMD at the hip in the crude and adjusted models (OR = 3.14, 95% CI: 1.55–6.38 and OR = 2.35, 95% CI: 1.01–5.46, respectively); however, the association was no longer significant after adjusting for additional covariates (Table S10). When women were divided by menopausal status, the results were similar in postmenopausal and premenopausal groups; we observed a significant association with BMD as a continuous variable (Table S11). No association was observed in men with low hip BMD; however, in the adjusted model, we observed lower hip BMD in men carrying the TG-rs7041 genotype (Table S10).

### 3.4. Association between VDBP Levels and BMD

The Spearman correlation showed a positive, but weak, correlation between VDBP levels and hip BMD (r = 0.158, *p* < 0.001) and with femoral neck BMD (r = 0.140, *p* < 0.001). We also observed a weak correlation between total 25(OH)D levels and hip BMD (r = 0.083, *p* = 0.021), femoral neck BMD (r = 0.119, *p* = 0.0009) and lumbar spine BMD (r = 0.080, *p* = 0.025) in postmenopausal women (Table S12). However, no significant correlation was observed among men (Table S13).

After stratifying by hip BMD status, we found that the adjusted median of VDBP levels was lower in postmenopausal women with low hip BMD, compared to those with normal hip BMD. However, we did not observe statistically significant differences for free or bioavailable 25(OH)D between these groups (Table S14). Furthermore, no differences were observed in VDBP levels and VitD forms when stratified by femur and lumbar spine BMD status (Table S14).

We further investigated if serum VDBP levels could be associated with BMD. We observed a strong protective association between VDBP levels, in the highest category, against low BMD at the hip in the adjusted models for women (OR = 0.67; 95% CI: 0.47–0.96, *p* = 0.029, respectively) (Table S15). Since this association was only observed in women, we stratified by menopausal status. The same effect was observed in postmenopausal women in the highest VDBP category, compared to those in the lowest VDBP category (OR = 0.54; 95% CI: 0.33–0.77, *p* = 0.002). No significant association was found in premenopausal women (Table 2). Interestingly, when we stratified by VitD status, the association between VDBP and low BMD was significant only in VitD deficient individuals (OR = 0.60; 95% CI: 0.37–0.97, *p* = 0.038), regardless of other potential confounders such as age groups, sex, BMI categories, alcohol, leisure time physical activity, smoking and VitD intake (Table 3).

On the other hand, in linear regression analyses, VDBP levels were positively associated with hip and femoral neck BMD in all participants in the crude and adjusted models. When divided by sex, in women the association was observed only in the crude model at the hip, femoral neck and lumbar spine. On the other hand, in men the association was observed at the hip and femoral neck in the adjusted models (Table 4). Furthermore, we did not observe significant associations when we stratified by menopausal status or VitD status (Tables S16 and S17).

### 3.5. Association between Different Forms of VitD and BMD

We also evaluated the relationship between the different forms of VitD (total, free and bioavailable 25(OH)D) and BMD at different body regions, but we did not observe a statistically significant association, except for total VitD and BMD, of the femoral neck in all participants (β 0.02, 95% CI: 0.002–0.04, *p* = 0.027) and in women (β 0.02, 95% CI: 0.0003 –0.04, *p* = 0.047) (Tables S18–S20).

### 3.6. Interactions between VDBP Levels and VitD Intake in Low-Hip BMD

We found significant interactions between VitD intake and serum VDBP levels with respect to low hip BMD (p_interaction_ = 0.045). Women in the highest categories of VitD intake and VDBP levels had a lower risk of having low BMD at the hip than women in the lowest categories (Table 5). There was no significant interaction between VitD intake and VDBP with BMD at other sites (lumbar spine or femoral neck) in men, premenopausal or postmenopausal women (p_interaction_ > 0.05). Furthermore, we did not observe interactions between other nutrients and VDBP for low BMD at the hip, femoral neck or lumbar spine (p_interaction_ > 0.05).

## 4. Discussion

Up to date, there is no evaluation of the association between variants of the *GC* gene, serum VDBP levels, the different forms of VitD and BMD in the Mexican population. Our study shows data regarding the effect of the main haplotypes and respective alleles of the *GC* gene on serum VDBP concentrations and BMD in the Mexican population. First, we observed significant differences in serum VDBP concentration between women and men, being lower in men. These results are similar to those reported by Bolland et al. (339 vs. 305 μmol/L, *p* = 0.005) [12]. Afterwards, we found that serum VDBP concentration significantly diminishes with aging in women but not in men. Previous evidence has suggested that exposure to estrogen can increase the levels of VDBP, whereas androgen does not affect VDBP concentration [3]. 

We further investigate if the *GC* variants and haplotypes had an influence on the serum VDBP concentration. The C and G alleles of rs4588 and rs7041, respectively, were significantly more frequent within the highest tertil of VDBP levels in women and men. When we looked at the haplotypes, serum VDBP concentration was significantly lower in heterozygous GC2/GC1S and GC2/GC1F compared to individual GC1S/GC1S. Interestingly, serum VDBP concentration in GC2/GC2 women was not statistically significant different compared to carriers of two GC1 haplotypes. It has been proposed that differences in concentration can be related to differential synthesis rate or half-life of the variants [22]. Our data support an effect of the *GC* variants on serum VDBP concentration. Nevertheless, it is important to bear in mind other influential factors such as ethnicity, estrogen, cytokines, liver conditions and steroids [23].

We then analyzed the relationship of serum VDBP and total, free or bioavailable 25(OH)D concentrations with BMD. We did not observe significant differences of total 25(OH)D between the lowest and highest tertiles of VDBP in both women and men. Previous studies regarding the correlation of total, free or bioavailable 25(OH)D with VDBP levels have shown inconsistent results [12,24,25,26,27]. Our data showed a negative correlation of VDBP levels with free and bioavailable 25(OH)D after SNP adjustment (rho −0.349, *p* < 0.001 and rho −0.344, *p* < 0.001, respectively). However, no correlation was observed with total 25(OH)D (rho = 0.008, *p* = 0.735); these results were similar when stratifying by sex. The correlations between VDBP with free and bioavailable 25(OH)D not adjusted for genotypes were in the same direction (rho −0.538, *p* < 0.001 and rho −0.526, *p* < 0.001, respectively). Inconsistencies of the results could be explained by genetic background or by measurement methods. 

Our study shows significant lower serum VDBP levels in women with diminished hip BMD, compared to women with normal BMD. Furthermore, in women, a negative association between VDBP levels and BMD status was found. Interestingly, the association was observed in the group of men in the adjusted model of the linear regression. Previous evidence has shown influence of sex steroids and VDBP levels. Estradiol has a direct correlation with VDBP [28,29], whereas testosterone does not seem to have a direct influence [30], even though VDBP has been previously associated with BMD in elderly men [30]. The influence of sex hormones can be involved in these findings; a caveat is that we do not have dates regarding the years of menopause or estradiol levels for all women; therefore, interpretation should be cautious. Our results are consistent with previous findings suggesting that low serum VDBP levels are associated with low BMD and osteoporotic fracture in Mexican postmenopausal women and other populations [11,31,32]. Importantly, the association remained significant, for total hip BMD, when the analysis was adjusted by confounders. In other populations, lower concentrations of VDBP have also been found in osteoporotic postmenopausal women when compared to nonosteoporotic women [32]. In addition, we observed that high levels of VitD intake along with high levels of VDBP have a protective effect on BMD at the hip. Such an effect was not observed at the femoral neck or lumbar spine. These observations are in agreement with a recent meta-analysis demonstrating a beneficial effect of VitD supplements and enriched foods, along with calcium, on BMD and reduction in fragility fractures in postmenopausal women [33].

The effect of the *GC* SNPs, individually and as a haplotype, on BMD was mainly observed in women. Women carrying at least one copy of the A allele of rs4588 showed a significant reduction in BMD at the hip. It is important to mention that only a few homozygous AA individuals could be recruited due to the low frequency of this allele in our population. This could explain why the homozygous A had a slightly higher BMD at the hip in postmenopausal compared to premenopausal groups. Regarding rs7041, the G allele confers a higher BMD at the hip in both groups of women. Interestingly, the same alleles conferring higher levels of VDBP were also associated with higher BMD. On the other hand, in the group of women the carriers of at least one GC2 (rs7041-T/rs4588-A) haplotype showed lower BMD at the femoral neck and hip compared to noncarriers of GC2 (rs7041-T/rs4588-A), while at the lumbar spine only GC2/GC2 women has a reduced BMD. When we stratify women in pre- and postmenopausal groups, the GC1S/GC1S displayed the highest BMD at the hip, femoral neck and lumbar spine in both subgroups. This observation is congruent considering that GC1 haplotype contains the two alleles associated with the highest BMD (rs4588-C/rs7041-G). BMD at the hip significantly decreases in the presence of one copy of the GC2 (rs7041-T/rs4588-A) haplotype in the premenopausal group; this effect did not reach significance in the postmenopausal group. The lack of statistical significance could be related to the small number of homozygous GC2 (rs7041-T/rs4588-A) derived from the low frequency of the rs4588 A allele.

Our results support the influence of the *GC* genotypes on serum VDBP concentration and BMD. It has been difficult to fully understand the relationship between VDBP variants and BMD because results are not uniform between populations. In early postmenopausal women, the BMD mean at different anatomic regions did not differ between the three main *GC* haplotypes [6]. In postmenopausal women, the GC1 haplotype seems to be associated with increased fracture risk, while the GC2 (rs7041-T/rs4588-A) carriers were at minor risk. Interestingly, fracture risk association was independent of BMD [34].

Another important factor to consider is the serum concentration of total 25(OH)D, which did not show a significant difference between the groups of low and high VDBP. In European and Asian populations, the GC1 (either 1F (rs7041-T/rs4588-C) or 1S (rs7041-G/rs4588-C)) haplotypes have been associated with increased levels of 25(OH)D and 1,25(OH2)D, while GC2 (rs7041-T/rs4588-A) displays the opposite effect [35,36]. These differences in serum levels of VitD metabolites are expected to have an impact on bone health. It is important to bear in mind that the found associations have been proved to be influenced by other genetic variants, such as VitD Receptor (*VDR* gene) SNPs and environmental factors such as calcium and VitD intake. Additionally, other VDBP SNPs have also been associated with BMD, which adds more factors to the relationship [37]. 

Recently, it has been suggested that the estimates of free and bioavailable VitD may have better correlation with BMD [18,38]. A study in postmenopausal women showed an association between free and bioavailable VitD with hip and total body BMD only in women not taking calcium or VitD supplements [18]. In our study, we did not observe association or correlation between free or bioavailable 25(OH)D SNP adjusted and BMD, even when we stratified by calcium intake. A previous study showed that the monoclonal or polyclonal antibodies for the estimation of VDBP, along with ethnicity, could produce discrepancies in the association between the levels of free and bioavailable VitD and BMD at different sites. The association between free and bioavailable VitD was observed only with the monoclonal method in an African population; however, the estimates of free and bioavailable VitD did not consider the affinities of the *GC* gene variants [39]. Nevertheless, longitudinal studies are necessary to explore these associations.

VDBP can exert an effect on BMD through a different mechanism: it can serve as a reservoir for VitD metabolites. Considering this function, VDBP could regulate bioavailability and half-life of VitD metabolites. In our population, increased levels of VDBP seem to protect against BMD loss. On the other hand, VDBP can incentivize osteoclast function by being converted into the vitamin D-binding protein-macrophage activating factor (DBP-MAF) [40]. No other possible function of VDBP seems to have a known impact on BMD [1,41]. An interesting theory has been proposed to explain how higher levels of VDBP can be related to low BMD. This theory proposes that when VDBP is abundant it can be metabolized into the DBP-MAF, leading to increased bone resorption [35]. 

In this study, we stratified by sex to address its importance as a biological variable; however, additional studies in different populations are needed to determine if the observed association with serum VDBP levels and BMD are population-specific and the clinical relevance according to ethnicity.

Although we found statistically significant associations, this study has some limitations. First, VDBP measurement using monoclonal antibodies by ELISA could underestimate VDBP concentrations. The polymorphism of VDBP could challenge its accurate measurement, and therefore the results should be interpreted with caution. Second, the free and bioavailable 25(OH)D measurements were estimated from calculations based on a model development by Bikle et al. [42] and not directly. Unfortunately, the direct quantification of these metabolites is technically complicated by the small percentage of free and bioavailable 25(OH)D in serum (~0.03%), making it more difficult to measure. In addition, assays to directly measure free 25(OH)D and VDBP using a mass-spectrometry assay are not routinely applied in clinical practice and have been tested in small sample sizes or selected populations. In the future, these new methods could be applied to routine clinical samples to more accurately assess VitD status. Third, estrogen levels have been reported to influence VBDP levels [23,28]; our estimates of free and bioavailable VitD did not consider estrogen levels, and therefore this may lead to measurement errors. Fourth, we could not achieve population stratification due to the lack of ancestry estimates in the overall HWCS participants. However, only those individuals whose ancestors were born in the central region of Mexico (Cuernavaca, Morelos), for at least three generations, were included in the study. The strengths of our study are the analysis of a large group of individuals compared to other observational studies. Moreover, this cohort has been characterized in detail in previous publications. Second, the statistical analysis was rigorous, including adjusting for multiple confounding variables, reducing the risk of biased results. Third, this is the first effort to understand the effect of *GC* variants on serum VDBP levels, the different forms of VitD and BMD, in a Latin American population.

## 5. Conclusions

In conclusion, our results support an association of the two main *GC* variants with VDBP serum concentration and BMD in Mexican Mestizo women. High serum VDBP levels act as a protective factor against low BMD, mainly at the hip in postmenopausal women. The ultimate effect of the *GC* variants on VDBP and BMD could be influenced by several factors: the half-life of the different isoforms of VDBP, the affinity of each variant for VitD metabolites and the conversion rate towards DBP-MAF for each variant.

## Figures and Tables

**Table 1:** Demographics in categories of VDBP in both sex of individuals who belongs to the Health Workers Cohort Study.

	Women				Men			
	VDBP Levels				VDBP Levels			
Characteristics	Low ^c^ (106.8–250.8) * *n* = 430	Medium (250.9–303.9) * *n* = 430	High (304.2–449.7) * *n* = 429	*p* Value **	Low ^c^ (114.6–239.3) * *n* = 188	Medium (239.9–287.9) * *n* = 188	High (288.0–423.9) * *n* = 188	*p* Value **
Age (years) ^a^	55.5 (20)	53.0 (21)	53.0 (19)	<0.001	48.0 (22.5)	44.5 (19)	47.0 (20.5)	0.162
Leisure time physical activity (min/day) ^a^	9.6 (28.6)	9.6 (28.6)	9.6 (26.8)	0.493	12.9 (43.9)	19.1 (42.6)	14.2 (28.6)	0.433
Active (≥30 min/day), %	32.1	30.5	31.5	0.850	38.8	45.7	41.5	0.593
Smoking status								
Current, %	10.2	7.2	8.1	0.286	21.7	22.3	19.8	0.650
Past, %	22.9	22.5	25.7	0.175	38.6	36.2	44.5	0.246
BMI (kg/m^2^) ^a^	26.8 (5.9)	26.7 (6.0)	26.9 (6.4)	0.493	26.5 (4.8)	26.6 (4.3)	26.4 (5.7)	0.433
Overweight, %	48.9	51.1	45.2	0.277	41.6	38.5	39.6	0.693
Obesity, %	20.7	17.6	18.1	0.335	25.8	25.9	27.0	0.792
Body fat proportion ^a^	45.3 (8.6)	45.0 (8.4)	44.9 (8.1)	0.322	31.5 (6.9)	31.4 (6.7)	31.2 (8.3)	0.116
Femoral neck BMD ^b^, g/cm^2^	0.906 (0.156)	0.931 (0.139)	0.947 (0.135)	<0.001	1.012 (0.152)	1.042 (0.141)	1.035 (0.169)	0.464
Femur Low BMD, %	47.2	43.0	36.6	0.002	29.4	22.6	29.6	0.966
Hip BMD ^b^, g/cm^2^	0.948 (0.163)	0.967 (0.135)	0.985 (0.129)	<0.001	1.064 (0.140)	1.085 (0.132)	1.083 (0.158)	0.550
Hip Low BMD, %	34.7	27.0	22.1	<0.001	17.0	16.0	20.2	0.425
Lumbar spine BMD ^b^, g/cm^2^	1.053 (0.174)	1.064 (0.161)	1.071 (0.155)	0.243	1.147 (0.165)	1.151 (0.1444)	1.162 (0.162)	1.000
Lumbar spine Low BMD, %	54.8	53.9	51.5	0.333	48.4	41.5	40.1	0.106
Total 25-hydroxivitamin D (ng/mL)^a^	20.6 (7.4)	21.2 (8.3)	20.8 (8.4)	0.198	22.3 (8.5)	22.1 (8.7)	21.8 (8.7)	0.460
Free 25-hydroxivitamin D (pg/mL) ^a^	7.5 (2.5)	5.8 (2.2)	4.5 (2.1)	<0.001	7.5 (3.0)	6.3 (2.3)	5.1 (2.5)	<0.001
Bioavailable 25-hydroxivitamin D (ng/mL) ^a^	2.6 (1.1)	2.2 (0.9)	1.7 (0.8)	<0.001	2.9 (1.3)	2.4 (1.0)	1.9 (0.9)	<0.001
Free 25-hydroxivitamin D-SNP adjusted (pg/mL) ^a^	7.3 (4.3)	6.6 (3.9)	5.3 (3.5)	<0.001	8.1	7.3	5.9	<0.001
Bioavailable 25-hydroxivitamin D-SNP adjusted (ng/mL) ^a^	2.7 (1.6)	2.5 (1.5)	1.9 (1.3)	<0.001	3.1	2.8	2.3	<0.001
Vitamin D intake (UI/day) ^a^	150.3 (166.0)	147.4 (166.6)	133.1 (166.6)	0.092	141.7	155.3	140.5	0.335
Albumin (g/dL) ^a^	4.2 (0.4)	4.2 (0.4)	4.2 (0.4)	1.000	4.3 (0.4)	4.3 (0.4)	4.3 (0.5)	1.000
Alcohol (g/day) ^a^	0.43 (1.8)	0.58 (1.8)	0.79 (1.5)	0.011	2.2	3.1	2.7	0.051
rs4588								
C, n (%)	621 (72.9)	676 (80.0)	706 (84.0)	<0.001	279 (75.4)	298 (81.9)	310 (83.3)	0.009
A, n (%)	231 (27.0)	168 (20.0)	134 (16.0)	<0.001	91 (24.6)	66 (18.1)	62 (16.6)	0.007
CC, n (%)	214 (50.2)	266 (63.0)	293 (69.8)	<0.001	101 (54.6)	120 (65.9)	128 (68.8)	0.005
CA+AA, n (%)	212 (49.8)	156 (37.0)	127 (30.2)	<0.001	84 (45.4)	62 (34.1)	58 (31.2)	0.015
rs7041								
T, n (%)	475 (55.8)	455 (53.9)	395 (46.9)	0.0003	214 (57.8)	186 (50.8)	178 (47.9)	0.003
G, n (%)	377 (44.3)	389 (46.1)	447 (53.1)	0.0003	156 (42.2)	180 (49.2)	194 (52.2)	0.024
TT, n (%)	138 (32.4)	124 (29.4)	93 (22.1)	<0.001	63 (34.1)	44 (24.1)	37 (19.9)	0.002
TG, n (%)	199 (46.7)	207 (49.1)	209 (49.6)	0.415	88 (47.6)	97 (53.3)	104 (55.9)	0.110
GG, n (%)	89 (20.9)	91 (21.6)	119 (28.3)	0.011	34 (18.4)	41 (22.5)	45 (24.2)	0.173

* Minimum–maximum value of each category. ** Low vs. high categories. ^a^ Median (Interquartile range). ^b^ Mean (Standard deviation). ^c^ Low, medium and high nutrient category levels were defined by tertiles.

**Table 2:** Association between VDBP categories and low hip BMD by sex.

	Premenopausal Women, *n* = 513					Postmenopausal Women, *n* = 776					Men, *n* = 564				
	VDBP Tertiles					VDBP Tertiles					VDBP Tertiles				
	Low (128.6–255.9) *	Medium (256.0–308.9) *		High (309.0–449.0) *		Low (106.8–250.8) *	Medium (250.9–303.9) *		High (304.2–449.7) *		Low ^a^ (114.6–239.3) *	Medium ^a^ (239.9–287.9) *		High ^a^ (288.0–423.9) *	
Model	OR (95% CI)	OR (95% CI)	*p* Value	OR (95% CI)	*p* Value	OR (95% CI)	OR (95% CI)	*p* Value	OR (95% CI)	*p* Value	OR (95% CI)	OR (95% CI)		OR (95% CI)	*p* Value
Crude	Ref.	1.06 (0.55, 2.03)	0.868	1.48 (0.80, 2.74)	0.215	Ref.	0.67 (0.47, 0.96)	0.027	0.42 (0.29, 0.61)	<0.001	Ref.	0.926 (0.54, 1.60)	0.781	1.25 (0.73, 2.08)	0.427
Adjusted ^1^	Ref.	1.06 (0.53, 2.15)	0.860	1.41 (0.72, 2.79)	0.318	Ref.	0.76 (0.51, 1.14)	0.109	0.51 (0.34, 0.78)	0.002	Ref.	1.05 (0.57, 1.91)	0.884	1.28 (0.72, 2.28)	0.402
Adjusted ^2^	Ref.	1.14 (0.55, 2.34)	0.742	1.43 (0.71, 2.87)	0.319	Ref.	0.74 (0.49, 1.12)	0.153	0.54 (0.33, 0.77)	0.002	Ref.	1.04 (0.57, 1.91)	0.892	1.24 (0.69, 2.21)	0.473

^1^ Model is adjusted for age groups, BMI (normal, overweight, obesity), alcohol (g/day), leisure time physical activity (active ≥ 30 min/day) and smoking (never, smoking, former smoker). ^2^ Model is adjusted for age groups, BMI (normal, overweight, obesity), alcohol (g/day), leisure time physical activity (active ≥ 30 min/day), smoking (never, current, past), VitD intake and 25-hydroxivitamin D levels. ^a^ Low, medium and high VDBP category levels were defined by tertiles. * Minimum–maximum value of each category.

**Table 3:** Association between VDBP categories and low hip BMD by VitD status.

	Normal Vitamin D, *n* = 1077					Deficiency Vitamin D, *n* = 775				
	VDBP Tertiles					VDBP Tertiles				
	Low (106.8–247.5) *	Medium (247.6–298.6) *		High (298.7–448.0) *		Low (114.6–242.9) *	Medium (243.0–301.2) *		High (301.9–449.7) *	
Model	OR (95% CI)	OR (95% CI)	*p* Value	OR (95% CI)	*p* Value	OR (95% CI)	OR (95% CI)	*p* Value	OR (95% CI)	*p* Value
Crude	Ref.	**0.67** **(0.48, 0.95)**	**0.024**	0.82 (0.58, 1.14)	0.233	Ref.	0.79 (0.54, 1.16)	0.237	**0.57** **(0.38, 0.85)**	**0.006**
Adjusted ^1^	Ref.	0.79 (0.53, 1.17)	0.234	0.94 (0.64, 1.40)	0.770	Ref.	0.87 (0.55, 1.38)	0.552	**0.61** **(0.38, 0.98)**	**0.042**
Adjusted ^2^	Ref.	0.79 (0.53, 1.18)	0.256	0.95 (0.64, 1.41)	0.792	Ref.	0.86 (0.54, 1.38)	0.536	**0.60** **(0.37, 0.97)**	**0.038**

^1^ Model is adjusted for age groups, sex, BMI (normal, overweight, obesity), alcohol (g/day), leisure time physical activity (active > 30 min/day) and smoking (never, smoking, former smoker). ^2^ Model is adjusted for age groups, sex, BMI (normal, overweight, obesity), alcohol (g/day), leisure time physical activity (active > 30 min/day), smoking (never, current, past) and VitD intake. A Low, medium and high VDBP category levels were defined by tertiles. * Minimum–maximum value of each category. In bold statistically significant associations.

**Table 4:** Association between VDBP levels and BMD at different body regions.

	All Participants, *n* = 1893						All Women, *n* = 1289						All Men, *n* = 564					
	Hip BMD (g/cm^2^)		Femoral Neck * (g/cm^2^)		Spine Lumbar * (g/cm^2^)		Hip BMD (g/cm^2^)		Femoral Neck * (g/cm^2^)		Spine Lumbar * (g/cm^2^)		Hip BMD (g/cm^2^)		Femoral Neck * (g/cm^2^)		Spine Lumbar * (g/cm^2^)	
Model	β (95% CI)	*p* Value	β (95% CI)	*p* Value	β (95% CI)	*p* Value	β (95% CI)	*p* Value	β (95% CI)	*p* Value	β (95% CI)	*p* Value	β (95% CI)	*p* Value	β (95% CI)	*p* Value	β (95% CI)	*p* Value
Crude	**0.04** **(0.008, 0.07)**	**0.012**	**0.05** **(0.02, 0.08)**	**0.001**	0.03 (−0.006, 0.06)	0.108	**0.06** **(0.02, 0.09)**	**0.001**	**0.07** **(0.03, 0.10)**	**<0.001**	**0.04** **(0.001, 0.08)**	**0.043**	0.04 (−0.01, 0.10)	0.138	0.05 (−0.01, 0.11)	0.078	0.04 (−0.02, 0.10)	0.217
Adjusted ^1^	**0.03** **(0.003, 0.05)**	**0.030**	**0.03** **(0.005, 0.05)**	**0.016**	0.02 (−0.01, 0.05)	0.235	0.01 (−0.02, 0.04)	0.388	0.01 (−0.01, 0.04)	0.260	−0.002 (−0.04, 0.03)	0.884	**0.06** **(0.01, 0.11)**	**0.029**	**0.06** **(0.01, 0.11)**	**0.016**	0.05 (−0.01, 0.11)	0.081
Adjusted ^2^	**0.03** **(0.002, 0.05)**	**0.031**	**0.03** **(0.005, 0.05)**	**0.016**	0.02 (−0.01, 0.05)	0.214	0.01 (−0.02, 0.04)	0.415	0.02 (−0.01, 0.04)	0.259	−0.002 (−0.03, 0.03)	0.922	**0.06** **(0.007, 0.11)**	**0.025**	**0.06** **(0.01, 0.11)**	**0.014**	0.05 (−0.01, 0.11)	0.078

* VDBP levels were logarithmically transformed to the base 10. ^1^ Model is adjusted for age groups, sex, BMI (normal, overweight, obesity), alcohol (g/day), leisure time physical activity (active > 30 min/day) and smoking (never, smoking, former smoker). ^2^ Model is adjusted for age groups, sex, BMI (normal, overweight, obesity), alcohol (g/day), leisure time physical activity (active > 30 min/day), smoking (never, current, past), VitD intake and 25-hydroxivitamin D levels. In bold statistically significant associations.

**Table 5:** Association between VDBP and low hip BMD and its interaction with VitD intake quartiles in women (*n* = 1277).

		Women (*n* = 1277)				
		VDBP Levels				
		Low ^a^ (106.8–250.8)	Medium (250.9–303.9)		High (304.2–449.7)	
		Ref.	OR (95% CI)	*p* Value	OR (95% CI)	*p* Value
Vitamin D intake	Low ^b^ (1.5–85.9) *	1.0	1.55 (0.74–3.27)	0.247	1.50 (0.73–3.07)	0.274
	Medium (86.1–144.5) *	1.0	1.08 (0.53–2.18)	0.833	0.83 (0.40–1.74)	0.623
	High (144.7–253.1) *	1.0	0.37 (0.19–0.74)	0.004	0.39 (0.19–0.79)	0.009
	Very high (254.8–1275.6) *	1.0	0.80 (0.39–1.62)	0.538	0.44 (0.21–0.94)	0.035

The *p* -value of the Wald test was 0.045. Model 1 is adjusted for age groups, BMI (normal, overweight, obesity), alcohol (g/day), leisure time physical activity (active ≥ 30 min/day) and smoking (never, smoking, former smoker). ^a^ Low, medium and high VDBP category levels were defined by tertiles. ^b^ Low, medium, high and very high VDBP category levels were defined by quartiles. * Minimum–maximum value of each category.

## Data Availability

The data used to support the findings of this study are available from the corresponding author for anyone who requests it.

## Supplementary Materials

The following are available online at https://www.mdpi.com/article/10.3390/genes12081176/s1, Figure S1: Comparison of VDBP levels by GC gene variants and sex. (A) VDBP levels in men carrying rs4588 locus genotypes, (B) VDBP levels in women carrying rs4588 locus genotypes, (C) VDBP levels in men carrying rs7041 locus genotypes, (D) VDBP levels in women carrying rs7041 locus genotypes, (E) VDBP levels in men carrying GC diplotypes and (F) VDBP levels in women carrying GC diplotypes, Traditional nomenclature referring to the protein phenotypes: (a) GC1F (rs7041-T/rs4588-C); (b) GC1S (rs7041-G/rs4588-C) and (c) GC2 (rs7041-T/rs4588-A), Figure S2. Comparison of femoral neck BMD by GC gene variants and menopausal status. (A) Femoral neck BMD in premenopausal women carrying rs4588 locus genotypes, (B) femoral neck BMD in postmenopausal women carrying rs4588 locus genotypes, (C) femoral neck BMD in premenopausal women carrying rs7041 locus genotypes, (D) femoral neck BMD in postmenopausal women carrying rs7041 locus genotypes, (E) femoral neck BMD in premenopausal women carrying GC diplotypes and (F) femoral neck BMD in postmenopausal women carrying GC diplotypes. Traditional nomenclature referring to the protein phenotypes: (a) GC1F (rs7041-T/rs4588-C); (b) GC1S (rs7041-G/rs4588-C) and (c) GC2 (rs7041-T/rs4588-A), Figure S3. Comparison of lumbar spine BMD by GC gene variants and menopausal status. (A) Lumbar spine BMD in premenopausal women carrying rs4588 locus genotypes, (B) lumbar spine BMD in postmenopausal women carrying rs4588 locus genotypes, (C) lumbar spine BMD in premenopausal women carrying rs7041 locus genotypes, (D) lumbar spine BMD in postmenopausal women carrying rs7041 locus genotypes, (E) lumbar spine BMD in premenopausal women carrying GC diplotypes and (F) lumbar spine BMD in postmenopausal women carrying GC diplotypes. Traditional nomenclature referring to the protein phenotypes: (a) GC1F (rs7041-T/rs4588-C); (b) GC1S (rs7041-G/rs4588-C) and (c) GC2 (rs7041-T/rs4588-A), Table S1. Descriptive statistics of vitamin D metabolites by sex, Table S2. Factors associated with VDBP levels in all participants, Table S3. Factors associated with VDBP levels in all men, Table S4. Factors associated with VDBP levels in all women, Table S5. Factors associated with VDBP levels in premenopausal women, Table S6. Factors associated with VDBP levels in postmenopausal women, Table S7. Factors associated with VDBP levels in normal Vitamin D levels, Table S8. Factors associated with VDBP levels in deficiency Vitamin D levels, Table S9. Associations between GC gene variants and hip BMD in all participants, Table S10. Associations between GC gene variants and hip BMD by sex, Table S11. Associations between GC gene variants and low hip BMD by status menopausal, Table S12. Spearman correlation between VDBP levels, different forms of 25 hydroxyvitamin D and bone mineral density by menopausal status, Table S13. Spearman correlation between VDBP levels, different forms of 25 hydroxyvitamin D and bone mineral density by sex, Table S14. Descriptive statistics by sex and BMD status, Table S15. Association between VDBP categories and low hip BMD in all participants, Table S16. Association between VDBP levels and BMD by menopausal status, Table S17. Association between VDBP levels and BMD by vitamin D status, Table S18. Association between Vitamin D levels and BMD in all population, Table S19. Association between Vitamin D levels and BMD by sex, Table S20. Association between vitamin D levels and BMD by menopausal status.
