# Supplementary material for: Association of GC Variants with Bone Mineral Density and Serum VDBP Concentrations in Mexican Population
Source: Genes (Basel). 2021 Jul 29;12(8):1176. doi: 10.3390/genes12081176 (PMC8391993; doi:10.3390/genes12081176)
Supplement: Supplementary file 1 [file genes-12-01176-s001.zip › Supplementary Figures.pdf]

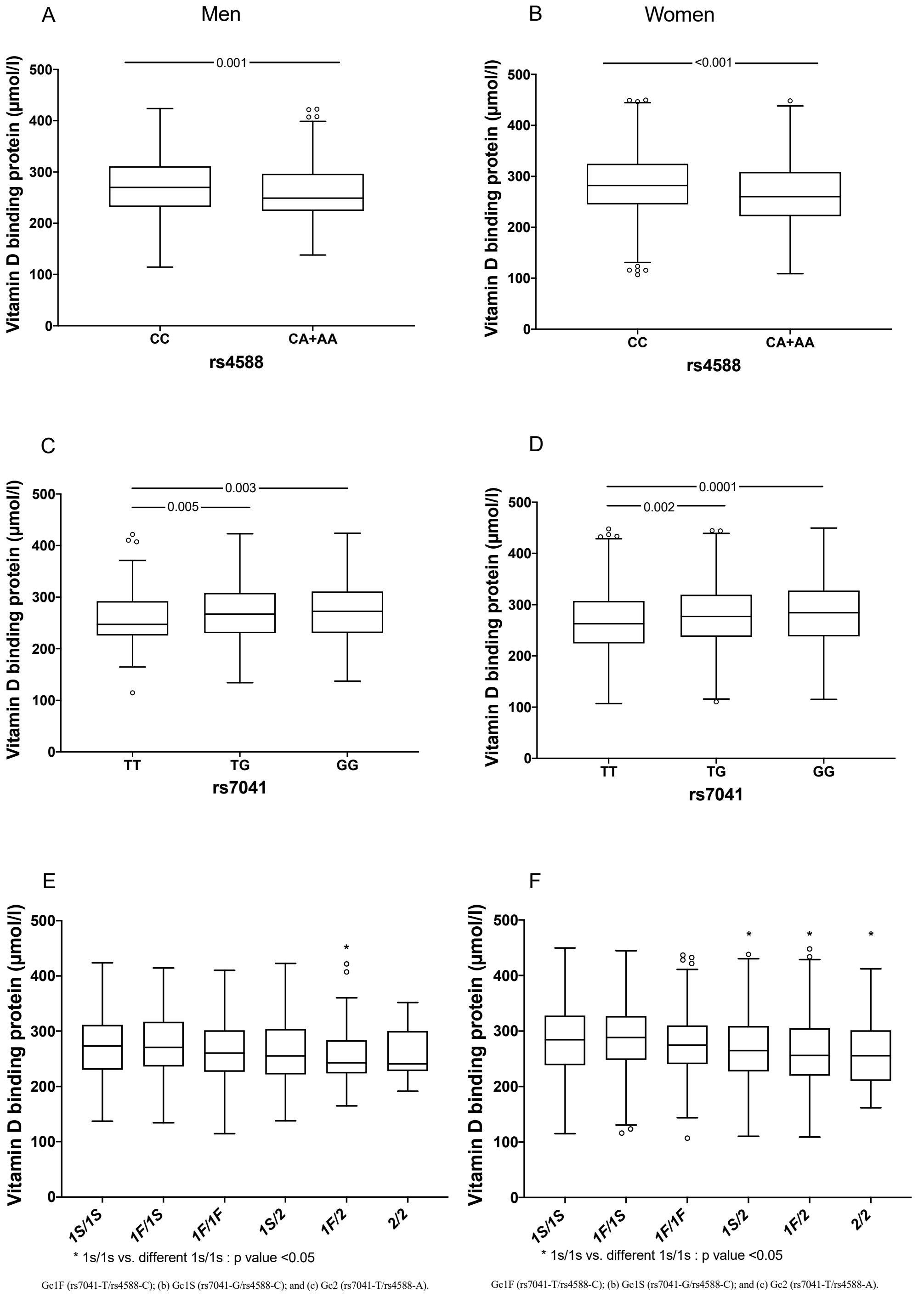

Figure S1. Comparison of VDBP levels by GC gene variants and sex. (A) VDBP levels in men carrying of rs4588 locus genotypes, (B) VDBP levels in women carrying of rs4588 locus genotypes, (C) VDBP levels in men carrying of rs7041 locus genotypes, (D) VDBP levels in women carrying of rs7041 locus genotypes, (E) VDBP levels in men carrying of GC haplotypes and (F) VDBP levels in women carrying of GC haplotypes. Traditional nomenclature referring to the protein phenotypes: (a) GC1F (rs7041-T/rs4588-C); (b) GC1S (rs7041-G/rs4588-C) and (c) GC2 (rs7041-T/rs4588-A).

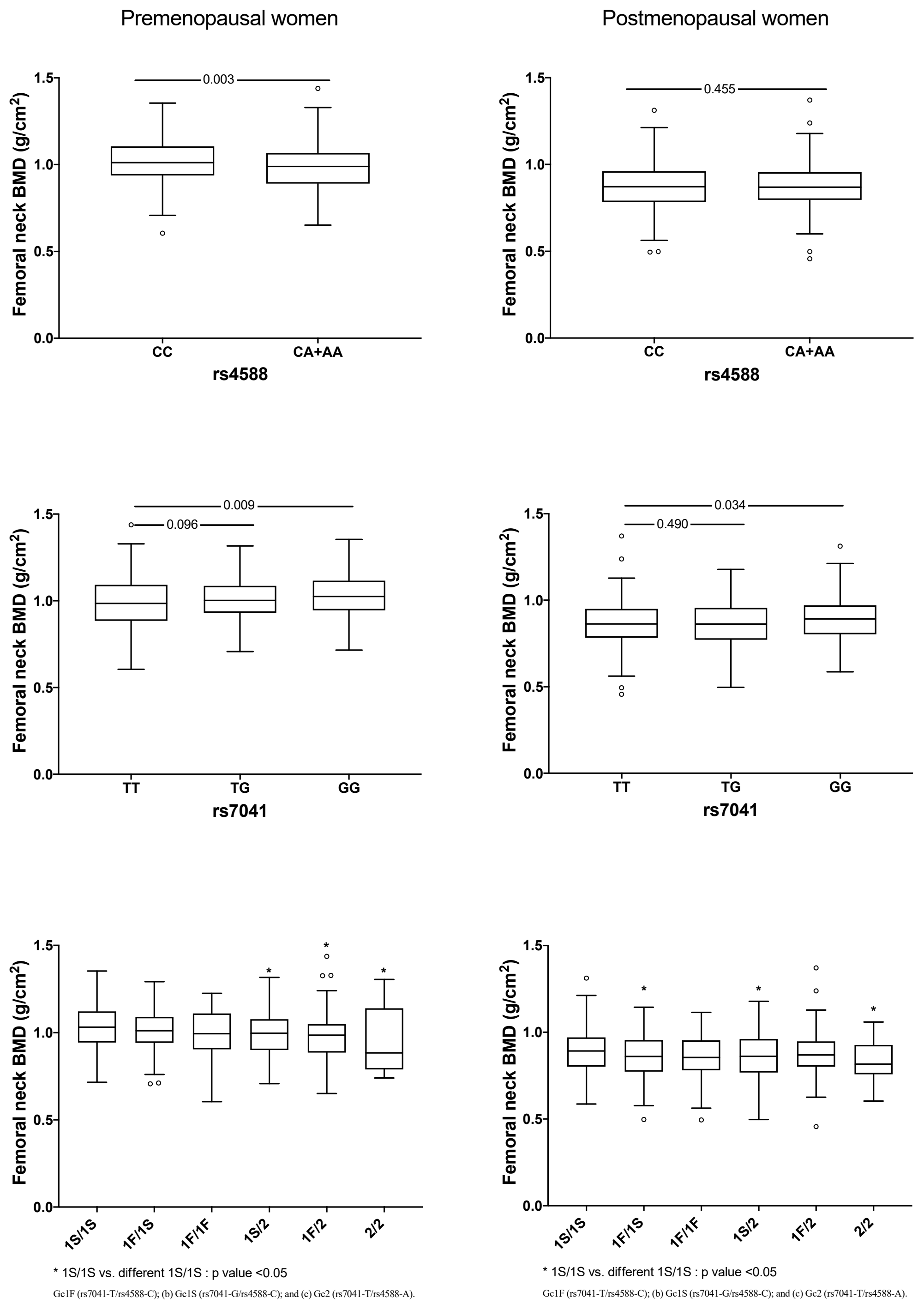

Figure S2. Comparison of femoral neck BMD by *GC* gene variants and menopausal status. (A) femoral neck BMD in premenopausal women carrying of rs4588 locus genotypes, (B) femoral neck BMD in postmenopausal women carrying of rs4588 locus genotypes, (C) femoral neck BMD in premenopausal women carrying of rs7041 locus genotypes, (D) femoral neck BMD in postmenopausal women carrying of rs7041 locus genotypes, (E) femoral neck BMD in premenopausal women carrying of GC haplotypes and (F) femoral neck BMD in postmenopausal women carrying of GC haplotypes. Traditional nomenclature referring to the protein phenotypes: (a) GC1F (rs7041-T/rs4588-C); (b) GC1S (rs7041-G/rs4588-C) and (c) GC2 (rs7041-T/rs4588-A).

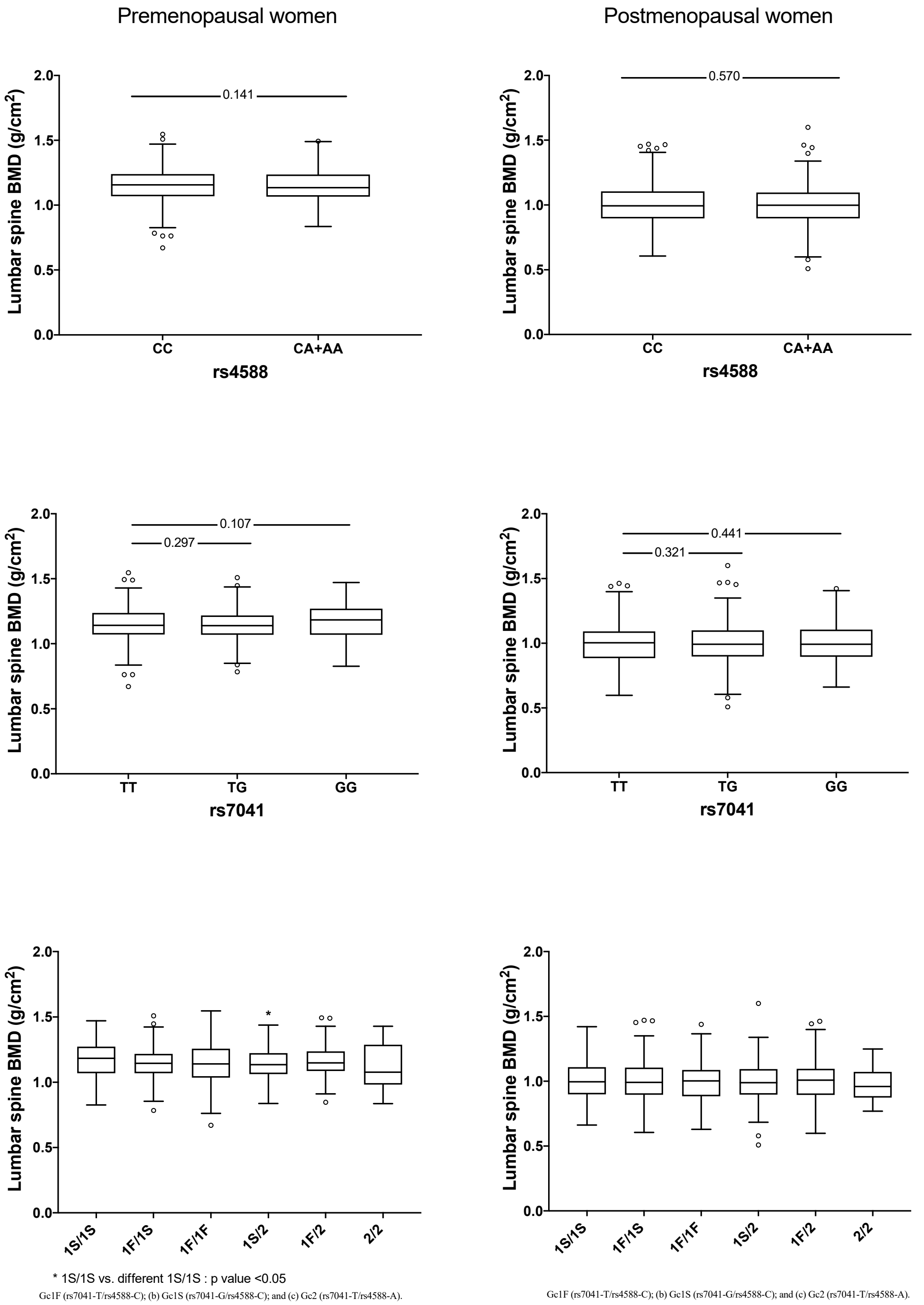

Figure S3. Comparison of lumbar spine BMD by *GC* gene variants and menopausal status. (A) lumbar spine BMD in premenopausal women carrying of rs4588 locus genotypes, (B) lumbar spine BMD in postmenopausal women carrying of rs4588 locus genotypes, (C) lumbar spine BMD in premenopausal women carrying of rs7041 locus genotypes, (D) lumbar spine BMD in postmenopausal women carrying of rs7041 locus genotypes, (E) lumbar spine BMD in premenopausal women carrying of GC haplotypes and (F) lumbar spine BMD in postmenopausal women carrying of GC haplotypes. Traditional nomenclature referring to the protein phenotypes: (a) GC1F (rs7041-T/rs4588-C); (b) GC1S (rs7041-G/rs4588-C) and (c) GC2 (rs7041-T/rs4588-A).
