# Supplementary material for: Association of GC Variants with Bone Mineral Density and Serum VDBP Concentrations in Mexican Population
Source: Genes (Basel). 2021 Jul 29;12(8):1176. doi: 10.3390/genes12081176 (PMC8391993; doi:10.3390/genes12081176)
Supplement: Supplementary file 1 [file genes-12-01176-s001.zip › Supplementary_tables.pdf]

Table S1. Descriptive statistics of VitD metabolites by sex.

Characteristics	All participants n=1,853	Women n=1289	Men n=565	*P* value
Total 25-hydroxivitamin D (ng/mL) [a]	21.1(20.8-21.5)	20.8(20.4-21.2)	21.9(21.3-22.5)	0.040
Vitamin D-binding protein (µmol/l) [a]	271.5(268.0-275.0)	275.1(270.9-279.3)	263.2(256.9-269.5)	0.001
Free 25-hydroxivitamin D (pg/mL) [a]	5.9(5.7-6.0)	5.7(5.6-5.8)	6.2(6.0-6.5)	0.001
Bioavailable 25-hydroxivitamin D (ng/mL) [a]	2.2(2.2-2.3)	2.1(2.0-2.2)	2.4(2.3-2.5)	<0.001
Free 25-hydroxivitamin D-SNP adjusted (pg/mL) [a]	6.7(6.5-6.9)	6.5(6.3-6.7)	7.2(6.9-7.5)	<0.001
Bioavailable 25-hydroxivitamin D-SNP adjusted (ng/mL) [a]	2.5(2.5-2.6)	2.4(2.3-2.5)	2.7(2.6-2.8)	<0.001

[a] Adjusted median (95% CI). Median adjusted by age groups, BMI (normal, overweight, obesity), leisure time physical activity (active >150 min/week), vitamin D intake (quartiles) and smoking (never, smoking, former smoker).

Table S2. Factors associated with VDBP levels in all participants.

Variable	Univariable model Coefficient (95% CI)					Multivariable model Coefficient (95% CI)				
	10^{h}	25th	50th	75th	90th	10^{h}	25th	50th	75th	90th
Age(years)	**-0.6* (-0.9,-0.4)**	**-0.5* (-0.7,-0.3)**	**-0.3* (-0.6,-0.1)**	-0.1 (-0.4,0.2)	**-0.5* (-0.9,-0.02)**	**-0.6* (-0.9,-0.2)**	**-0.7* (-1.0,-0.4)**	**-0.5* (-0.8,-0.2)**	-0.2 (-0.6,0.2)	-0.2 (-0.8,0.3)
Leisure time physical activity										
Inactive (<30 min/day)	Ref.	Ref.	Ref.	Ref.	Ref.	Ref.	Ref.	Ref.	Ref.	Ref.
Active (≥30 min/day)	-1.5 (-10.7,7.6)	6.9 (-0.6,14.5)	**11.6* (3.9,19.3)**	**15.0* (5.9,24.1)**	**18.8* (4.2,33.3)**	9.1 (-1.46,19.7)	8.7 (-1.0,18.4)	**13.1* (3.4,22.8)**	**15.0* (2.2,27.8)**	**19.5* (2.7,36.3)**
Smoking status	**-22.4* (-32.4,-12.4)**	**-12.2* (-22.3,-2.2)**	-7.5 (-16.9,1.8)	-7.5 (-17.6,2.7)	**-23.1* (-37.0,-9.3)**	-7.3 (-19.2,4.5)	3.5 (-7.4,14.4)	1.6 (-9.3,12.4)	-0.6 (-14.9,13.8)	-5.9 (-24.8,12.9)
Normal										
Current	Ref.	Ref.	Ref.	Ref.	Ref.	Ref.	Ref.	Ref.	Ref.	Ref.
Past	1.6 (-7.4,10.5)	-2.5 (-9.9,4.9)	-6.11 (-13.6,1.33)	-1.11 (-9.9,7.7)	-8.9 (-24.6,6.8)	0.11 (-8.1,8.3)	-4.6 (-12.1,3.0)	-7.1 (-14.6,0.4)	-4.2 (-14.2,5.7)	-8.3 (-21.3,4.8)
BMI (kg/m^2)										
Normal	Ref.	Ref.	Ref.	Ref.	Ref.	Ref.	Ref.	Ref.	Ref.	Ref.
Overweight	-2.1 (-15.1,11.0)	-2.3 (-13.5,8.8)	-10.1 (-21.1,0.8)	-2.5 (-16.2,11.1)	-14.4 (-35.5,6.6)	-1.1 (-13.3,11.2)	-8.0 (-19.3,3.2)	-6.4 (-17.6,4.8)	1.2 (-13.6,16.1)	-4.8 (-24.3,14.6)
Obesity	-2.5 (-12.1,7.0)	0.5 (-7.6,8.6)	-0.07 (-8.1,8.0)	2.9 (-7.1,12.9)	3.2 (-12.2,18.5)	-2.4 (-11.2,6.5)	1.6 (-6.6,9.7)	3.1 (-5.0,11.3)	5.1 (-5.6,15.9)	7.4 (-6.7,21.6)
Total 25-hydroxivitamin D (ng/mL)[a]										
Vitamin D intake (UI/day) [a]	Ref.	Ref.	Ref.	Ref.	Ref.	Ref.	Ref.	Ref.	Ref.	Ref.
Alcohol(g/day) [a]	-4.3 (-13.7,5.0)	-8.1 (-15.5,-0.6)	-7.3 (-15.5,0.9)	-3.3 (-12.9,6.2)	-12.5 (-27.5,2.4)	-1.81 (-10.6,7.0)	-4.5 (-12.6,3.6)	0.6 (-7.4,8.7)	-1.6 (-12.3,9.1)	-7.2 (-21.2,6.7)
rs4588	-6.5 (-17.3,4.3)	-5.9 (-14.5,2.8)	-3.7 (-13.1,5.7)	-5.9 (-17.0,5.2)	-5.6 (-22.9,11.8)	-9.2 (-19.4,1.1)	-7.0 (-16.5,2.4)	0.8 (-8.6,10.2)	-3.0 (-15.5,9.4)	-9.2 (-25.5,7.2)
CC	0.5 (-0.1,1.1)	0.4 (-0.1,0.9)	0.1 (-0.4,0.6)	-0.3 (-0.9,0.3)	**-1.3* (-2.3,-0.3)**	0.4 (-0.2,1.0)	0.3 (-16.5,2.4)	0.04 (-0.5,0.6)	-0.7 (-1.4,0.004)	**-1.1* (-2.1,-0.2)**
CA+AA	-0.0001 (-0.02,0.02)	-0.001 (-0.02,0.02)	-0.002 (-0.02,0.02)	-0.008 (-0.03,0.02)	-0.002 (-0.04,0.04)	-0.003 (-0.02,0.02)	-0.007 (-0.03,0.01)	0.002 (-0.02,0.02)	0.005 (-0.02,0.03)	-0.002 (-0.04,0.03)
rs7041	0.05 (-0.20,0.30)	0.02 (-0.19,0.22)	-0.05 (-0.26,0.16)	-0.10 (-0.35,0.16)	-0.004 (-0.43,0.42)	0.05 (-0.2,0.3)	-0.008 (-0.22,0.20)	-0.03 (-0.24,0.18)	0.23 (-0.04,0.51)	0.22 (-0.14,0.58)
TT										
TG	Ref.	Ref.	Ref.	Ref.	Ref.	Ref.	Ref.	Ref.	Ref.	Ref.
GG	**-8.6* (-16.2,-1.0)**	**-16.7* (-22.8,-10.6)**	**-20.3* (-26.1,-14.4)**	**-16.5* (-23.5,-9.5)**	**-17.2* (-29.1,-5.3)**	**-9.9* (-17.6,-2.2)**	**-14.9* (-21.9,-7.8)**	**-18.3* (-25.4,-11.3)**	**-15.7* (-25.0,-6.4)**	-9.1 (-21.3,3.1)
Age(years)										
Leisure time physical activity	Ref.	Ref.	Ref.	Ref.	Ref.	Ref.	Ref.	Ref.	Ref.	Ref.
Inactive (<30 min/day)	2.9 (-7.3,13.0)	**10.8* (2.3,19.2)**	**15.3* (7.4,23.2)**	**15.6* (6.0,25.3)**	**17.3* (2.3,32.3)**	0.8 (-8.4,10.0)	7.7 (-0.8,16.2)	7.1 (-1.3,15.6)	9.5 (-1.7,20.7)	7.0 (-7.7,21.7)
Active (≥30 min/day)	5.9 (-6.2,17.9)	**11.7* (1.7,21.7)**	**22.2* (12.8,31.6)**	**22.1* (10.6,33.6)**	**32.7* (14.8,50.5)**	-2.8 (-14.6,9.0)	1.4 (-9.4,12.3)	7.5 (-3.4,18.3)	12.0 (-2.3,26.3)	15.8 (-3.0,34.6)

*P value <0.05

Table S3. Factors associated with VDBP levels in all men.

Variable	Univariable model					Multivariable model				
	Coefficient (95% CI)					Coefficient (95% CI)				
	10^{h}	25th	50th	75th	90th	10^{h}	25th	50th	75th	90th
Age(years)	**-0.4***<br>**(-0.8,-0.02)**	**-0.4***<br>**(-0.7,0.02)**	**-0.2**<br>**(-0.7,0.2)**	0.4<br>(-0.2,0.9)	0.3<br>(-0.5,1.2)	-0.4<br>(-0.8,0.01)	**-0.5***<br>**(-0.8,-0.1)**	-0.05<br>(-0.5,0.3)	0.2<br>(-0.4,0.8)	0.6<br>(-0.2,1.3)
Leisure time physical activity										
Inactive (<30 min/day)	Ref.	Ref.	Ref.	Ref.	Ref.	Ref.	Ref.	Ref.	Ref.	Ref.
Active (≥30 min/day)	0.4<br>(-13.0,13.8)	2.2<br>(-9.1,13.5)	-4.5<br>(-17.7,8.6)	-6.1<br>(-22.8,10.6)	-8.0<br>(-29.2,13.3)	2.7<br>(-10.0,15.4)	-3.0<br>(-14.2,8.2)	-1.9<br>(-13.8,10.1)	-5.5<br>(-23.6,12.6)	-6.4<br>(-28.5,15.7)
Smoking status										
Normal	Ref.	Ref.	Ref.	Ref.	Ref.	Ref.	Ref.	Ref.	Ref.	Ref.
Current	7.7<br>(-10.5,25.9)	-1.9<br>(-12.7,16.6)	3.4<br>(-13.7,20.4)	2.5<br>(-19.1,24.2)	6.4<br>(-22.1,35.0)	-1.3<br>(-17.9,15.2)	1.4<br>(-13.2,16.0)	5.9<br>(-9.6,21.5)	4.5<br>(-19.0,28.1)	13.4<br>(-15.3,42.1)
Past	-8.5<br>(-23.7,6.7)	-1.5<br>(-13.7,10.7)	9.3<br>(-5.0,23.6)	12.0<br>(-6.1,30.2)	**24.4***<br>**(0.5,48.3)**	-7.2<br>(-21.1,6.8)	2.3<br>(-10.0,14.6)	7.6<br>(-5.5,20.8)	8.4<br>(-11.5,28.3)	31.0<br>(6.7,55.2)
BMI (kg/m^2)										
Normal	Ref.	Ref.	Ref.	Ref.	Ref.	Ref.	Ref.	Ref.	Ref.	Ref.
Overweight	3.9<br>(-8.6,16.4)	-3.4<br>(-15.3,8.5)	**-15.5***<br>**(-28.4,-2.7)**	-13.2<br>(-31.2,4.9)	-6.0<br>(-35.0,23.1)	3.7<br>(-10.1,17.6)	-3.5<br>(-15.7,8.7)	-11.4<br>(-24.4,1.6)	-11.0<br>(-30.7,8.8)	-18.6<br>(-42.7,5.5)
Obesity	-12.2<br>(-28.2,3.8)	-15.6<br>(-30.8,-0.4)	-11.9<br>(-28.3,4.6)	-20.7<br>(-43.8,2.3)	-16.2<br>(-53.4,20.9)	-13.1<br>(-30.9,4.6)	**-21.8***<br>**(-37.4,-6.1)**	-12.8<br>(-29.5,3.9)	-23.0<br>(-48.3,2.3)	-33.0<br>(63.9,-2.1)
Total 25-hydroxivitamin D (ng/mL)[a]	0.3<br>(-0.7,1.3)	0.1<br>(-0.6,0.9)	0.2<br>(-0.8,1.1)	-0.5<br>(-1.6,0.7)	-0.5<br>(-2.2,1.1)	0.2<br>(-0.7,1.1)	-0.2<br>(-1.0,0.6)	-0.4<br>(-1.3,0.5)	-0.9<br>(-2.3,0.4)	-0.9<br>(-2.5,0.7)
Vitamin D intake (UI/day) [a]	-0.0004<br>(-0.03,0.03)	0.001<br>(-0.02,0.03)	0.01<br>(-0.02,0.04)	0.02<br>(-0.02,0.05)	0.002<br>(-0.05,0.05)	-0.003<br>(-0.03,0.03)	-0.006<br>(-0.03,0.02)	0.009<br>(-0.02,0.04)	0.02<br>(-0.02,0.06)	0.004<br>(-0.05,0.05)
Alcohol(g/day) [a]	0.003<br>(-0.23,0.24)	0.02<br>(-0.17,0.21)	-0.04<br>(-0.27,0.19)	0.25<br>(-0.05,0.55)	0.30<br>(-0.08,0.67)	0.05<br>(-0.16,0.26)	0.02<br>(-0.17,0.20)	-0.04<br>(-0.24,0.16)	-0.02<br>(-0.32,0.28)	0.07<br>(-0.29,0.44)
rs4588										
CC	Ref.	Ref.	Ref.	Ref.	Ref.	Ref.	Ref.	Ref.	Ref.	Ref.
CA+AA	**-11.1***<br>**(-22.0,-0.19)**	-7.6<br>(-17.5,2-4)	**-17.5***<br>**(-28.0,-6.9)**	-12.5<br>(-28.3,3.4)	-9.9<br>(-31.6,11.8)	-9.0<br>(-21.9,3.9)	-10.1<br>(-21.5,1.3)	-11.2<br>(-23.4,0.9)	-14.6<br>(-33.0,3.8)	-13.4<br>(-35.9,9.0)
rs7041										
TT	Ref.	Ref.	Ref.	Ref.	Ref.	Ref.	Ref.	Ref.	Ref.	Ref.
TG	2.9<br>(-12.1,18.0)	4.9<br>(-8.3,18.1)	**20.1***<br>**(6.1,34.2)**	16.1<br>(-4.0,36.3)	12.6<br>(-20.7,45.9)	4.5<br>(-11.0,19.9)	4.0<br>(-9.6,17.6)	12.5<br>(-2.0,27.1)	15.9<br>(-6.2,37.9)	13.2<br>(-13.6,40.1)
GG	9.4<br>(-8.8,27.7)	6.3<br>(-9.7,22.3)	**25.3***<br>**(8.3,42.3)**	18.6<br>(-5.9,43.0)	16.7<br>(-23.6,57.1)	2.7<br>(-17.4,22.7)	4.1<br>(13.6,21.8)	**19.9***<br>**(1.0,38.8)**	14.9<br>(-13.7,43.5)	19.5<br>(-15.4,54.5)

*P value <0.05

Table S4. Factors associated with VDBP levels in all women.

Variable	Univariable model<br>Coefficient (95% CI)					Multivariable model<br>Coefficient (95% CI)				
	10^{h}	25th	50th	75th	90th	10^{h}	25th	50th	75th	90th
Age(years)	**-0.8***<br>**(-1.1,-0.5)**	**-0.7***<br>**(-1.0,-0.4)**	**-0.6***<br>**(-0.8,-0.3)**	**-0.4***<br>**(-0.7,-0.03)**	**-0.9***<br>**(-1.4,-0.4)**	**-0.76***<br>**(-1.3,-0.2)**	**-0.7***<br>**(-1.2,0.2)**	**-1.0***<br>**(-1.4,-0.5)**	**-0.6***<br>**(-1.2,-0.1)**	**-1.3***<br>**(-2.0,0.7)**
Leisure time physical activity	**-16.1***<br>**(-25.9,-6.3)**	-8.6<br>(-18.4,1.3)	-5.8<br>(-14.9,3.3)	-6.4<br>(-16.4,3.4)	**-23.1***<br>**(-36.8,-9.4)**	-3.8<br>(-19.5,11.9)	6.4<br>(-7.8,20.7)	12-4<br>(-0.4,25.3)	6.0<br>(-11.1,23.1)	12.9<br>(-6.8,32.6)
Inactive (<30 min/day)										
Active (≥30 min/day)	Ref.	Ref.	Ref.	Ref.	Ref.	Ref.	Ref.	Ref.	Ref.	Ref.
Smoking status	0.4<br>(-12.3,13.1)	-6.5<br>(-16.8,3.8)	-6.0<br>(-15.8,3.8)	1.1<br>(-8.8,11.1)	-6.6<br>(-25.2,12.0)	0.9<br>(-10.4,12.2)	-4.2<br>(-14.4,6.1)	-6.1<br>(-15.3,3.1)	-1.5<br>(-13.7,10.8)	-3.2<br>(-17.4,10.9)
Normal										
Current	Ref.	Ref.	Ref.	Ref.	Ref.	Ref.	Ref.	Ref.	Ref.	Ref.
Past	-7.0<br>(-27.7,13.7)	-1.7<br>(-19.5,16.1)	-14.2<br>(-30.4,2.0)	-2.3<br>(-19.9,15.3)	-12.6<br>(-41.2,16.0)	-2.2<br>(-21.3,16.9)	-8.8<br>(-25.9,8.9)	-17.5<br>(-33.1,1.8)	-1.6<br>(-22.5,19.2)	2.4<br>(-21.6,26.4)
BMI (kg/m^2)	-0.4<br>(-14.0,13.2)	1.1<br>(-10.5,12.8)	0.9<br>(-9.7,11.5)	2.6<br>(-8.9,14.2)	-4.0<br>(-22.7,14.8)	0.5<br>(-11.8,12.9)	1.6<br>(-9.6,12.9)	-1.0<br>(-11.9,9.1)	1.3<br>(-12.1,14.8)	-3.2<br>(-17.4,10.9)
Normal										
Overweight	Ref.	Ref.	Ref.	Ref.	Ref.	Ref.	Ref.	Ref.	Ref.	Ref.
Obesity	-11.2<br>(-23.5,1.0)	-10.7<br>(-21.4,0.01)	-3.9<br>(-14.4,6.7)	-0.5<br>(-11.5,10.5)	-15.3<br>(-32.9,2.3)	-5.3<br>(-17.4,6.7)	-4.6<br>(-15.6,6.4)	2.7<br>(-7.2,12.6)	3.9<br>(-9.3,17.0)	5.6<br>(-9.6,20.7)
Total 25-hydroxivitamin D (ng/mL)[a]	-3.8<br>(-17.4,9.8)	-2.2<br>(-14.1,9.7)	-0.4<br>(-12.1,11.4)	0.9<br>(-11.3,13.2)	-7.9<br>(-27.5,11.7)	-5.8<br>(-19.3,7.7)	-3.0<br>(-15.3,9.3)	1.5<br>(-9.5,12.6)	7.8<br>(-6.9,22.5)	3.5<br>(-13.5,20.4)
Vitamin D intake (UI/day) [a]	0.7<br>(-0.04,1.4)	0.6<br>(-0.9,1.3)	0.3<br>(-0.4,1.0)	-0.3<br>(-1.1,0.4)	**-1.5***<br>**(-2.7,-0.3)**	0.5<br>(-0.4,1.3)	0.4<br>(-0.4,1.1)	0.08<br>(-0.6,0.7)	-0.5<br>(-1.3,0.4)	-1.2<br>(-2.3,-0.2)
Alcohol(g/day) [a]	0.004<br>(-0.03,0.04)	-0.01<br>(-0.04,0.02)	-0.003<br>(-0.03,0.03)	-0.02<br>(-0.05,0.01)	-0.03<br>(-0.09.0.02)	0.02<br>(-0.009,0.06)	-0.007<br>(-0.04,0.02)	-0.01<br>(-0.04,0.01)	-0.02<br>(-0.05,0.02)	-0.009<br>(-0.05,0.03)
rs4588	0.07<br>(-1.2,1.3)	0.1<br>(-1.0,1.2)	-0.1<br>(-1.1,0.8)	-0.2<br>(-1.2,0.9)	-0.3<br>(-2.1,1.6)	0.3<br>(-0.9,1.4)	0.13<br>(-0.9,1.2)	0.4<br>(-0.5,1.4)	-0.5<br>(-1.7,0.8)	-0.45<br>(-1.9,1.0)
CC										
CA+AA	Ref.	Ref.	Ref.	Ref.	Ref.	Ref.	Ref.	Ref.	Ref.	Ref.
rs7041	-8.4<br>(-19.4,2.6)	**-20.4***<br>**(-27.9,-12.9)**	**-19.4***<br>**(-27.1,-11.7)**	**-15.7***<br>**(-24.4,-6.9)**	**-19.2***<br>**(-33.4,5.0)**	**-10.8***<br>**(-21.0,-0.6)**	**-17.5***<br>**(-26.7,-8.2)**	**-18.7***<br>**(-27.0,-10.3)**	**-12.8***<br>**(-23.9,-1.7)**	**-13.6***<br>**(-26.5,-0.82)**
TT										
TG	Ref.	Ref.	Ref.	Ref.	Ref.	Ref.	Ref.	Ref.	Ref.	Ref.
GG	1.9<br>(-10.8,14.6)	**12.9***<br>**(2.2,23.6)**	**14.4***<br>**(4.5,24.4)**	**12.3***<br>**(0.6,24.1)**	14.0<br>(-3.4,31.4)	-5.1<br>(-17.4,7.2)	5.5<br>(-5.7,16.7)	5.8<br>(-4.3,15.9)	10.0<br>(-3.4,23.4)	2.4<br>(-13.1,17.8)
Age(years)	0.1<br>(-14.9,15.1)	**13.9***<br>**(1.3,26.5)**	**21.5***<br>**(9.8,33.2)**	**20.5***<br>**(6.7,34.4)**	**32.1***<br>**(11.6,53.6)**	-10.9<br>(-26.5,4.6)	-2.2<br>(-16.4,11.9)	6.7<br>(-6.0,19.4)	14.4<br>(-2.5,31.4)	18.1<br>(-1.5,37.6)

*P value <0.05

Table S5. Factors associated with VDBP levels in premenopausal women.

Variable	Univariable model					Multivariable model				
	Coefficient (95% CI)					Coefficient (95% CI)				
	10^{h}	25th	50th	75th	90th	10^{h}	25th	50th	75th	90th
Age(years)	**-0.9* (-1.5,0.2)**	**-0.9* (-1.5,-0.3)**	**-0.9* (-1.5,-0.3)**	0.03 (-0.8,0.9)	-0.4 (-1.3,0.6)	**-1.0* (-1.7,-0.2)**	**-0.7* (-1.3,-0.1)**	**-0.07* (-1.3,-0.1)**	-0.3 (-1.0,0.6)	-0.2 (-1.3,0.9)
Leisure time physical activity										
Inactive (<30 min/day)	Ref.	Ref.	Ref.	Ref.	Ref.	Ref.	Ref.	Ref.	Ref.	Ref.
Active (≥30 min/day)	-0.9 (-17.3,15.6)	2.9 (-12.7,18.5)	1.8 (-13.8,17.4)	2.7 (-16.1,21.5)	8.7 (-14.3,31.8)	-1.8 (-20.0,16.3)	2.7 (-12.1,17.5)	5.6 (-8.8,19.9)	1.01 (-18.2,20.2)	-3.4 (-29.9,23.1)
Smoking status										
Normal	Ref.	Ref.	Ref.	Ref.	Ref.	Ref.	Ref.	Ref.	Ref.	Ref.
Current	-6.0 (-28.2,16.2)	-1.1 (-18.7,16.4)	7.9 (-9.2,24.9)	3.2 (-17.1,23.5)	4.2 (-23.9,32.3)	-8.4 (-38.4,21.6)	-12.4 (-36.9,11.9)	-19.9 (-18.6,14.2)	13.6 (-18.1,45.3)	11.0 (-32.7,54.8)
Past	4.8 (-24.8,34.4)	-3.8 (-27.3,19.7)	-11.1 (-34.0,11.6)	18.5 (-8.6,45.6)	-16.2 (-53.8,21.3)	-7.5 (-28.3,13.4)	-6.0 (-23.0,10.9)	-2.2 (-43.5,3.8)	-5.4 (-27.4,16.6)	-1.5 (-31.9,28.9)
BMI (kg/m^2)										
Normal	Ref.	Ref.	Ref.	Ref.	Ref.	Ref.	Ref.	Ref.	Ref.	Ref.
Overweight	0.9 (-15.3,17.1)	-7.4 (-21.4,6.5)	-14.8 (-30.5,0.9)	-7.8 (-28.9,13.3)	-27.4* (-52.1,-2.7)	3.7 (-15.7,23.1)	-3.8 (-19.6,12.0)	-1.3 (-16.6,14.0)	-5.8 (-26.3,14.7)	-26.2 (-54.5,2.0)
Obesity	-17.1 (-36.2,2.1)	**-20.7* (-37.2,-4.2)**	**-23.4* (-42.0,-4.8)**	0.8 (-24.2,25.8)	-5.3 (-34.5,24.0)	-3.6 (-26.4,19.2)	-9.1 (-27.7,9.4)	-16.0 (-34.0,2.0)	0.7 (-23.4,24.8)	-8.7 (-41.9,24.6)
Total 25-hydroxivitamin D (ng/mL)[a]	0.6 (-0.6,1.9)	0.6 (-0.5,1.7)	-0.1 (-1.3,1.0)	-0.9 (-2.4,0.6)	**-1.9* (-3.5,-0.3)**	0.6 (-0.7,2.0)	0.2 (-1.0,1.3)	0.05 (-1.0,1.1)	**-1.9* (-3.3,-0.4)**	**-2.5* (-4.5,-0.5)**
Vitamin D intake (UI/day) [a]	-0.04 (-0.10,0.02)	-0.01 (-0.06,0.04)	0.002 (-0.05,0.05)	-0.03 (-0.09,0.03)	-0.04 (-0.12,0.03)	-0.03 (-0.09,0.03)	-0.01 (-0.05,0.04)	-0.03 (-0.07,0.02)	-0.03 (-0.1,0.03)	-0.08 (-0.16,0.01)
Alcohol(g/day) [a]	0.5 (-1.2,2.2)	0.7 (-0.8,2.2)	0.1 (-1.5,1.6)	0.6 (-1.3,2.4)	-0.02 (-2.4,2.4)	1.2 (-0.7,3.1)	1.3 (-0.3,2.8)	1.2 (-0.3,2.7)	0.9 (-1.1,2.9)	1.0 (-1.8,3.7)
rs4588										
CC	Ref.	Ref.	Ref.	Ref.	Ref.	Ref.	Ref.	Ref.	Ref.	Ref.
CA+AA	**-18.0* (-33.8,-2.1)**	**-23.6* (-35.1,-12.1)**	**-22.7* (-35.0,-10.3)**	-16.0 (-33.5,1.6)	-6.7 (-26.6,13.2)	-15.1 (-32.1,1.9)	**-19.4* (-33.2,-5.6)**	**-19.3* (-32.7,-5.9)**	-13.8 (-31.8,4.2)	-3.1 (-27.9,21.7)
rs7041										
TT	Ref.	Ref.	Ref.	Ref.	Ref.	Ref.	Ref.	Ref.	Ref.	Ref.
TG	4.3 (-15.6,24.1)	**17.0* (1.3,32.6)**	**24.3* (7.3,41.3)**	**23.0* (2.1,43.9)**	**37.3* (12.3,62.2)**	-2.2 (-22.3,17.9)	7.9 (-8.5,24.3)	10.2 (-5.6,26.1)	19.6 (-1.7,40.9)	**32.3* (3.0,61.7)**
GG	10.8 (-13.5,35.2)	**19.3* (0.1,38.5)**	**31.8* (10.8,52.7)**	**28.2* (2.6,53.9)**	**41.4* (10.9,72.0)**	-8.9 (-35.0,17.2)	-0.004 (-21.2,21.2)	11.6 (-9.0,32.2)	25.0 (-2.7,52.6)	30.1 (-8.0,68.1)

*P value <0.05

Table S6. Factors associated with VDBP levels in post-menopausal women.

	Univariable model					Multivariable model				
	Coefficient (95% CI)					Coefficient (95% CI)				
Variable	10h	25th	50th	75th	90th	10h	25th	50th	75th	90th
Age(years)	-0.6 (-1.2,0.1)	**-0.9* (-1.6,-0.3)**	**-1.0* (-1.6,-0.3)**	**-1.2* (-1.9,-0.6)**	**-1.2* (-2.4,-0.1)**	-0.7 (-1.5,0.2)	**-0.9* (-1.6,-0.1)**	**-1.2* (-1.8,-0.6)**	**-1.4* (-2.2,-0.6)**	**-1.7* (-2.8,-0.7)**
Leisure time physical activity										
Inactive (<30 min/day)	Ref.	Ref.	Ref.	Ref.	Ref.	Ref.	Ref.	Ref.	Ref.	Ref.
Active (≥30 min/day)	3.1 (-9.8,16.1)	-11.2 (-26.2,3.8)	-9.9 (-22.4,2.7)	-0.7 (-14.9,13.6)	-2.7 (-24.2,18.8)	2.3 (-14.4,19.0)	-7.9 (-23.1,7.4)	**-18.3* (-30.8,-5.8)**	-8.9 (-25.0,7.2)	-11.1 (-31.8,9.6)
Smoking status										
Normal	Ref.	Ref.	Ref.	Ref.	Ref.	Ref.	Ref.	Ref.	Ref.	Ref.
Current	-23.6 (-46.1,1.9)	-15.2 (-42.7,12.3)	-16.8 (-39.5,6.0)	-4.3 (-29.9,21.3)	-12.5 (-53.5,28.5)	-21.3 (-50.3,7.6)	-4.0 (-20.4,12.5)	**-26.6* (-48.2,-4.9)**	-22.8 (-50.6,5.0)	-9.2 (-45.1,26.6)
Past	8.9 (-6.3,24.1)	7.4 (-9.0,23.9)	1.9 (-11.6,15.5)	1.8 (-13.4,17.1)	-2.1 (-26.6,22.4)	7.4 (-10.1,24.9)	5.1 (-12.9,23.2)	5.6 (-7.5,18.7)	2.5 (-14.3,19.3)	6.2 (-15.5,27.9)
BMI (kg/m^2)										
Normal	Ref.	Ref.	Ref.	Ref.	Ref.	Ref.	Ref.	Ref.	Ref.	Ref.
Overweight	-3.4 (-19.4,12.6)	-11.6 (-27.7,4.4)	2.3 (-12.4,17.0)	7.8 (-8.6,24.1)	9.6 (-13.5,32.8)	-15.0 (-33.0,3.0)	-4.0 (-20.4,12.5)	7.0 (-6.5,20.5)	14.6 (-2.7,32.0)	12.4 (-10.0,34.7)
Obesity	10.3 (-6.9,27.6)	6.7 (-7.6,27.0)	9.3 (-6.6,25.1)	6.8 (-10.9,24.4)	9.6 (-15.4,34.5)	-0.2 (-20.0,19.5)	5.1 (12.9,23.2)	8.8 (-6.0,23.6)	17.9 (-1.1,36.9)	7.0 (-17.5,31.4)
Total 25-hydroxivitamin D (ng/mL)[a]	0.9 (-0.1,1.9)	0.8 (-0.2,1.8)	0.6 (-0.3,1.5)	0.2 (-0.8,1.2)	-0.3 (-1.7,1.1)	0.6 (-0.6,1.7)	0.5 (-0.6,1.5)	0.6 (-0.3,1.4)	0.3 (-0.8,1.4)	-1.0 (-2.4,0.4)
Vitamin D intake (UI/day) [a]	0.02 (-0.02,0.06)	-0.01 (-0.05,0.03)	-0.003 (-0.04,0.03)	0.001 (-0.04,0.04)	-0.01 (-0.07,0.05)	0.04 (-0.007,0.09)	0.008 (-0.03,0.05)	0.006 (-0.03,0.04)	0.0002 (-0.04,0.05)	0.02 (-0.04,0.08)
Alcohol(g/day) [a]	0.007 (-1.4,1.4)	-0.44 (-2.0,1.1)	-0.34 (-1.7,1.0)	-0.69 (-2.1,0.7)	-1.5 (-3.6,0.5)	-0.9 (-2.6,0.7)	-0.07 (-1.6,1.4)	-0.1 (-1.4,1.1)	-0.7 (-2.3,0.9)	-1.7 (-3.7,0.4)
rs4588										
CC	Ref.	Ref.	Ref.	Ref.	Ref.	Ref.	Ref.	Ref.	Ref.	Ref.
CA+AA	-1.6 (-14.0,10.8)	**-18.3* (-28.7,-7.8)**	**-21.2* (-31.1,-11.3)**	**-15.1* (-26.2,-4.1)**	-16.2 (-32.7,0.31)	-11.0 (-25.5,3.6)	**-15.7* (-29.0,-2.4)**	**-20.3* (-31.2,-9.4)**	-13.4 (-27.5,0.6)	-15.9 (-22.9,2.2)
rs7041										
TT	Ref.	Ref.	Ref.	Ref.	Ref.	Ref.	Ref.	Ref.	Ref.	Ref.
TG	1.4 (-11.8,14.7)	9.7 (-6.4,25.8)	8.6 (-4.6,21.9)	4.0 (-10.8,18.7)	-0.7 (-23.6,22.1)	-4.1 (-21.9,13.7)	2.5 (-13.7,18.9)	3.2 (-10.1,16.6)	-3.9 (-21.1,13.2)	-4.7 (-26.8,17.3)
GG	-2.6 (-17.9,12.6)	15.9 (-2.6,34.4)	**17.0* (1.8,32.2)**	12.6 (-4.3,29.6)	16.4 (-9.9,42.7)	10.1 (-32.2,12.0)	2.9 (-17.3,23.0)	0.4 (-16.1,19.7)	0.7 (-20.6,21.9)	15.7 (-11.7,43.1)

*P value <0.05

Table S7. Factors associated with VDBP levels in normal VitD levels.

Variable	Univariable model Coefficient (95% CI)					Multivariable model Coefficient (95% CI)				
	10^{h}	25th	50th	75th	90th	10^{h}	25th	50th	75th	90th
Age(years)	**-0.6* (-1.0,-0.3)**	**-0.5* (-0.8,-0.2)**	**-0.4* (-0.7,0.1)**	-0.2 (-0.5,0.2)	-0.5 (-1.1,0.1)	-0.4 (-0.8,0.1)	**-0.4* (-0.8,-0.02)**	**-0.6* (-1.0,0.2)**	**-0.3 (-0.8,0.1)**	-0.5 (-1.1,0.1)
Sex										
Men	Ref.	Ref.	Ref.	Ref.	Ref.	Ref.	Ref.	Ref.	Ref.	Ref.
Women	2.4 (-8.5,13.2)	**9.6* (0.05,19.1)**	**12.6* (2.3,23.0)**	**18.1* (8.5,27.7)**	17.3 (-0.9,35.4)	**18.5* (3.3,33.7)**	**-0.4* (-0.8,-0.02)**	**-0.6* (-1.0,0.2)**	11.6 (-3.9,27.0)	15.4 (-3.0,33.9)
Menopause	**-15.3* (-25.1,-5.5)**	1.3 (-7.8,10.4)	4.9 (-5.0,14.9)	5.7 (-4.4,15.7)	-1.9 (-21.3,17.5)	**-22.6* (-39.6,-5.6)**	10.1 (-2.6,22.8)	**12.9* (0.5,25.3)**	7.3 (-10.0,24.6)	-0.09 (-20.7,20.6)
Leisure time physical activity										
Inactive (<30 min/day)	Ref.	Ref.	Ref.	Ref.	Ref.	Ref.	Ref.	Ref.	Ref.	Ref.
Active (≥30 min/day)	-0.9 (-11.8,10.1)	-4.1 (-13.3,5.0)	-8.5 (-18.3,1.2)	-2.9 (13.2,7.4)	-2.2 (-22.6,18.3)	-1.6 (-13.1,9.9)	0.2 (-13.4,5.8)	-8.5 (-17.8,0.9)	-0.7 (-12.4,11.0)	-7.6 (-21.5,6.3)
Smoking status										
Normal	Ref.	Ref.	Ref.	Ref.	Ref.	Ref.	Ref.	Ref.	Ref.	Ref.
Current	3.5 (-13.3,20.4)	2.4 (-11.2,16.0)	-11.4 (-25.7,2.8)	-1.4 (-17.2,14.4)	-8.5 (-38.4,21.3)	-2.7 (-19.6,14.2)	-3.0 (-17.2,11.1)	-5.1 (-18.9,8.6)	0.5 (-16.7,17.7)	-5.1 (-25.6,15.4)
Past	-2.6 (-15.2,10.0)	2.0 (-8.1,12.1)	-5.1 (-15.7,5.5)	2.1 (-9.7,13.8)	-2.7 (-24.9,19.5)	0.02 (-12.4,12.4)	-0.05 (-10.4,10.3)	1.8 (-8.4,11.9)	3.2 (-9.4,15.8)	-0.6 (-15.6,14.5)
BMI (kg/m^2)										
Normal	Ref.	Ref.	Ref.	Ref.	Ref.	Ref.	Ref.	Ref.	Ref.	Ref.
Overweight	-6.9 (-17.7,3.8)	-6.1 (-16.1,3.9)	-9.9 (-20.5,0.7)	-6.1 (-17.7,5.6)	-9.1 (-29.0,10.7)	2.6 (-9.8,14.9)	-5.1 (-15.5,5.2)	-1.1 (-11.1,9.0)	-5.1 (-17.6,7.5)	-7.4 (-22.4,7.5)
Obesity	-6.1 (-18.8,6.7)	-0.1 (-12.0,11.8)	-1.3 (-13.9,11.3)	-2.6 (-16.4,11.2)	-7.6 (-31.2,16.0)	-4.2 (-19.0,10.7)	-1.6 (-14.0,10.8)	1.8 (-10.2,13.9)	-3.5 (-18.6,11.5)	-8.1 (-26.1,9.9)
Total 25-hydroxivitamin D (ng/mL)[a]	0.03 (-1.0,1.01)	0.34 (-0.49,1.17)	0.28 (-0.62,1.18)	0.05 (-0.92,1.01)	-1.28 (-2.89,0.33)	-0.09 (-1.16,1.00)	0.27 (-0.62,1.16)	0.20 (-0.66,1.06)	-0.18 (-1.3,0.9)	-1.4 (-2.7,-0.1)*
Vitamin D intake (UI/day) [a]	0.0009 (-0.03,0.03)	0.003 (-0.02,0.03)	0.003 (-0.02,0.03)	0.003 (-0.02,0.03)	0.0002 (-0.05,0.05)	0.006 (-0.02,0.03)	0.003 (-0.02,0.03)	0.009 (-0.01,0.03)	0.005 (-0.02,0.03)	-0.005 (-0.04,0.03)
Alcohol(g/day) [a]	-0.02 (-0.3,0.3)	0.01 (-0.2,0.2)	-0.05 (-0.3,0.2)	-0.1 (-0.4,0.1)	-0.2 (-0.7,0.3)	0.05 (-0.22,0.31)	-0.008 (-0.23,0.21)	-0.07 (-0.28,0.15)	-0.10 (-0.37,0.16)	-0.10 (-0.42,0.22)
rs4588										
CC	Ref.	Ref.	Ref.	Ref.	Ref.	Ref.	Ref.	Ref.	Ref.	Ref.
CA+AA	-7.7 (-18.2,2.8)	**-14.2* (-22.7,-5.7)**	**-20.3* (-28.5,-12.0)**	**-14.8* (-24.4,-5.2)**	-14.5 (-29.8,0.8)	-10.7 (-21.9,0.5)	**-15.7* (-25.0,-6.3)**	**-19.3* (-28.4,-10.2)**	**-12.1* (-23.4,-0.7)**	-12.0 (-9.6,30.2)
rs7041										
TT	Ref.	Ref.	Ref.	Ref.	Ref.	Ref.	Ref.	Ref.	Ref.	Ref.
TG	5.8 (-6.6,18.2)	**14.7* (4.1,25.3)**	**17.9* (6.6,29.2)**	**15.0* (2.6,27.4)**	10.4 (-11.3,32.0)	5.4 (-8.0,18.8)	**12.2* (1.0,23.4)**	**11.9* (1.0,22.8)**	5.9 (-7.8,19.5)	2.4 (-13.8,18.7)
GG	1.7 (-12.7,16.0)	8.5 (-3.7,20.8)	**21.1* (8.0,341)**	**16.0* (1.7,30.4)**	23.0 (-2.0,48.1)	-5.3 (-21.7,11.1)	-1.1 (-14.8,12.7)	10.6 (-2.7.24.0)	7.6 (-9.1,24.3)	10.3 (-9.6,30.2)

*P value <0.05

Table S8. Factors associated with VDBP levels in deficiency VitD levels.

Variable	Univariable model					Multivariable model				
	Coefficient (95% CI)					Coefficient (95% CI)				
	10^{h}	25th	50th	75th	90th	10^{h}	25th	50th	75th	90th
Age(years)	**-0.7* (-1.1,-0.4)**	**-0.6* (-0.9,-0.3)**	**-0.3 (-0.6,0.1)**	-0.02 (-0.5,0.5)	-0.2 (-0.8,0.3)	**-0.9* (-1.5,-0.3)**	**-0.8* (-1.2,-0.3)**	-0.2 (-0.6,0.3)	-0.1 (-0.8,0.5)	0.05 (-0.7,0.8)
Sex										
Men	Ref.	Ref.	Ref.	Ref.	Ref.	Ref.	Ref.	Ref.	Ref.	Ref.
Women	-1.6 (-15.6,12.4)	2.1 (-10.4,14.5)	10.6 (-1.4,22,5)	9.5 (-7.4,26.3)	19.0 (-4.9,42.9)	8.0 (-11.8,27.8)	1.2 (-13.9,16.3)	12.8 (-2.6,28.3)	16.2 (-5.9,38.3)	**28.3* (3.0,53.7)**
Menopause	-7.8 (-21.4,5.8)	-6.7 (-17.7,4.3)	-0.6 (-11.5,10.3)	-5.9 (-20.9,9.1)	-13.8 (-31.1,3.4)	-1.2 (-23.3,21.0)	8.9 (-7.9,25.8)	-6.0 (-23.3,11.3)	-12.9 (-37.6,11.9)	-14.1 (-42.5,14.3)
Leisure time physical activity										
Inactive (<30 min/day)	Ref.	Ref.	Ref.	Ref.	Ref.	Ref.	Ref.	Ref.	Ref.	Ref.
Active (≥30 min/day)	1.8 (-11.8,15.5)	-3.4 (-15.6,8.8)	-0.9 (-12.4,10.6)	0.4 (-16.4,17.2)	-10.2 (-31.5,11.0)	1.7 (-14.2,17.5)	-0.9 (-12.9,11.2)	-4.0 (-16.4,8.4)	-0.5 (-18.2,17.2)	-0.6 (-20.9,19.8)
Smoking status										
Normal	Ref.	Ref.	Ref.	Ref.	Ref.	Ref.	Ref.	Ref.	Ref.	Ref.
Current	-3.7 (-24.9,17.6)	-4.5 (-22.8,13.7)	-13.8 (-31.3,3.8)	-2.4 (-27.6,22.8)	-16.2 (-50.4,18.0)	-9.5 (-33.9,15.0)	-9.4 (-28.0,9.2)	-11.8 (-30.9,7.3)	-1.1 (-22.4,20.1)	-7.2 (-38.5,24.2)
Past	-5.7 (-20.8,9.3)	-2.0 (-15.0,10.9)	4.1 (-8.4,16.5)	10.5 (-7.3,28.4)	1.1 (-23.1,25.4)	-3.6 (-20.8,13.5)	0.5 (-12.6,13.6)	5.5 (-7.8,19.0)	-1.8 (-20.8,17.2)	20.0 (-2.0,42.1)
BMI (kg/m^2)										
Normal	Ref.	Ref.	Ref.	Ref.	Ref.	Ref.	Ref.	Ref.	Ref.	Ref.
Overweight	-1.1 (-17.0,14.7)	-11.0 (-24.9,2.8)	-4.7 (-18.5,9.0)	-9.9 (-28.5,8.7)	-5.4 (-34.7,23.9)	-5.8 (-22.9,11.2)	-6.2 (-19.2,6.8)	-0.5 (-13.8,12.8)	-1.7 (-4.4,1.0)	-9.6 (-31.4,12.2)
Obesity	1.2 (-16.5,19.0)	-11.9 (-24.3,0.5)	-3.8 (-16.0,8.5)	0.3 (-16.3,16.9)	-9.6 (-35.8,16.6)	-13.0 (-32.0,6.0)	-12.1 (-26.6,2.4)	-8.6 (-23.4,6.3)	-1.1 (-22.4,20.1)	-4.4 (-28.8,20.0)
Total 25-hydroxivitamin D (ng/mL)[a]	1.1 (-1.0,3.3)	0.01 (-1.8,1.8)	-1.8 (-3.6,-0.05)	-1.8 (-4.3,0.8)	-2.8 (-6.0,0.4)	0.7 (-1.7,3.1)	-0.003 (-1.8,1.8)	-1.8 (-3.6,0.1)	-1.7 (-4.4,1.0)	-2.0 (-5.1,1.0)
Vitamin D intake (UI/day) [a]	-0.04 (-0.09,0.0001)	-0.02 (-0.05,0.02)	-0.02 (-0.05,0.02)	-0.02 (-0.07,0.03)	-0.003 (-0.08,0.07)	-0.002 (-0.05,0.05)	-0.005 (-0.04,0.03)	-0.02 (-0.06,0.02)	-0.04 (-0.1,0.01)	0.03 (-0.04,0.09)
Alcohol(g/day) [a]	0.2 (-0.4,0.9)	0.09 (-0.5,0.7)	**0.7* (0.2,1.3)**	0.4 (-0.4,1.2)	0.2 (-0.9,1.2)	0.4 (-0.4,1.2)	0.4 (-0.2,1.0)	**0.7* (0.1,1.3)**	0.4 (-0.4,1.3)	0.2 (-0.8,1.2)
rs4588										
CC	Ref.	Ref.	Ref.	Ref.	Ref.	Ref.	Ref.	Ref.	Ref.	Ref.
CA+AA	-9.1 (-21.6,3.4)	**-16.9* (-26.5,-7.2)**	**-20.4* (-30.1,-10.7)**	**-21.0* (-32.5,-9.5)**	**-20.1* (-36.5,-3.6)**	-10.0 (-24.2,4.2)	**-15.1* (-25.9,-4.3)**	**-15.3* (-26.4,-4.2)**	**-16.8* (-32.6,-1.0)**	-11.7 (-29.9,6.5)
rs7041										
TT	Ref.	Ref.	Ref.	Ref.	Ref.	Ref.	Ref.	Ref.	Ref.	Ref.
TG	2.6 (-16.7,11.5)	3.1 (-10.4,16.6)	**12.2* (0.1,24.2)**	**18.6* (1.3,36.0)**	**23.3* (3.5,43.2)**	-6.8 (-23.8,10.2)	-0.008 (-12.9,12.9)	4.4 (-8.9,17.6)	8.5 (-10.4,27.5)	15.8 (-6.0,37.6)
GG	8.4 (-9.1,25.9)	15.1 (-1.6,31.9)	**24.4* (9.4,39.4)**	**28.9* (7.4,50.5)**	**47.8* (23.1,72.5)**	-5.6 (-28.8,17.6)	6.7 (-11.0,24.4)	10.3 (-7.9,28.4)	17.4 (-8.4,43.3)	**39.3* (9.6,69.5)**

*P value <0.05

Table S9. Associations between *GC* gene variants and hip BMD in all participants.

		Categorical hip BMD				Continuous hip BMD			
	n	Crude model OR (95% CI)	*P* value	Adjusted model OR (95% CI)	*P* value	Crude model β (95% CI)	*P* value	Adjusted model β (95% CI)	*P* value
rs4588									
CC	1,122	Ref.		Ref.		Ref.		Ref.	
CA+AA	699	1.16(0.93,1.44)	0.178	1.14(0.88,1.46)	0.326	-0.02(-0.03,-0.001)	0.036	-0.01(-0.02,0.002)	0.138
rs7041									
TT	499	Ref.		Ref.		Ref.		Ref.	
TG	904	0.88(0.67,1.13)	0.321	0.87(0.65,1.16)	0.354	0.005(-0.01,0.02)	0.529	-0.0001(-0.01,0.01)	0.991
GG	419	0.79(0.58,1.07)	0.123	0.75(0.53,1.07)	0.113	0.01(-0.009,0.03)	0.273	0.01(-0.01,0.03)	0.196
Haplotype									
1S/1S	404	Ref.		Ref.		Ref.		Ref.	
1F/1S	537	1.05(0.77,1.42)	0.770	1.05(0.74,1.49)	0.796	0.0005(-0.02,0.02)	0.962	-0.005(-0.02,0.01)	0.529
1F/1F	181	1.04(0.69,1.58)	0.845	1.17(0.73,1.87)	0.521	-0.01(-0.04,0.02)	0.407	-0.02(-0.04,0.004)	0.119
1S/2	365	1.13(0.81,1.57)	0.479	1.17(0.80,1.71)	0.429	-0.019(-0.04,0.002)	0.081	**-0.02(-0.04,-0.002)**	**0.025**
1F/2	267	1.14(0.80,1.64)	0.467	1.14(0.75,4.82)	0.534	-0.008(-0.03,0.02)	0.503	-0.006(-0.03,0.01)	0.505
2/2	51	**2.75(1.51,5.00)**	**0.001**	**2.40(1.19,4.80)**	**0.014**	-0.04(-0.09,0.002)	0.059	-0.01(-0.05,0.02)	0.474

Model adjusted for age groups, sex, BMI (normal, overweight, obesity), alcohol (g/day), leisure time physical activity (active $\geq$150 min/week), smoking (never, current, past), vitamin D intake.
Traditional nomenclature referring to the protein phenotypes: (a) GC1F (rs7041-T/rs4588-C); (b) GC1S (rs7041-G/rs4588-C) and (c) GC2 (rs7041-T/rs4588-A).

Table S10. Associations between *GC* gene variants and hip BMD by sex.

	All women									All men								
		Categorical hip BMD				Continuous hip BMD					Categorical hip BMD				Continuous hip BMD			
		Crude model		Adjusted model		Crude model		Adjusted model			Crude model		Adjusted model		Crude model		Adjusted model	
	n	OR (95% CI)	*P* value	OR (95% CI)	*P* value	β (95% CI)	*P* value	β (95% CI)	*P* value	n	OR (95% CI)	*P* value	OR (95% CI)	*P* value	β (95% CI)	*P* value	β (95% CI)	*P* value
rs4588																		
CC	773	Ref.		Ref.		Ref.		Ref.		349	Ref.		Ref.		Ref.		Ref.	
CA+AA	495	1.10 (0.9,1.4)	0.426	1.08 (0.80,1.45)	0.629	-0.02 (-0.03,0.0001)	0.051	-0.009 (-0.02,0.004)	0.183	204	1.30 (0.83,2.04)	0.248	1.31 (0.79,2.17)	0.295	-0.01 (-0.03,0.02)	0.601	-0.01 (-0.03,0.02)	0.531
rs7041																		
TT	355	Ref.		Ref.		Ref.		Ref.		144	Ref.		Ref.		Ref.		Ref.	
TG	615	0.84 (0.63,1.12)	0.283	0.81 (0.58,1.14)	0.226	0.01 (-0.006,0.03)	0.186	0.01 (-0.01,0.03)	0.192	289	1.13 (0.65,1.96)	0.657	1.32 (0.72,2.43)	0.367	-0.02 (-0.05,0.005)	0.099	**-0.03 (-0.05,-0.001)**	**0.039**
GG	299	**0.66 (0.47,0.94)**	**0.021**	**0.62 (0.41,0.94)**	**0.025**	**0.03 (0.005,0.05)**	**0.018**	**0.02 (0.003,0.04)**	**0.021**	120	1.39 (0.73,2.62)	0.315	1.40 (0.69,2.83)	0.346	-0.03 (-0.06,0.008)	0.128	-0.02 (-0.05,0.01)	0.223
Haplotype																		
1S/1S	286	Ref.		Ref.		Ref.		Ref.		118	Ref.		Ref.		Ref.		Ref.	
1F/1S	353	1.26 (0.88,1.80)	0.202	1.23 (0.81,1.88)	0.333	-0.01 (-0.03,0.01)	0.285	-0.01 (-0.03,0.01)	0.443	184	0.67 (0.37,1.24)	0.201	0.76 (0.39,1.51)	0.439	0.01 (-0.02,0.05)	0.468	0.001 (-0.03,0.03)	0.933
1F/1F	134	1.29 (0.81.2.05)	0.277	1.47 (0.85,2.52)	0.167	-0.03 (-0.05,0.005)	0.099	**-0.03 (-0.05,-0.01)**	**0.015**	47	0.36 (0.12,1.11)	0.077	0.40 (0.12,1.31)	0.129	0.04 (-0.01,0.09)	0.123	0.02 (-0.02,0.07)	0.298
1S/2	260	1.16 (0.79,1.70)	0.454	1.17 (0.74,1.86)	0.493	-0.02 (-0.05,0.003)	0.079	-0.02 (-0.04,0.002)	0.073	105	1.04 (0.54,1.99)	0.910	1.28 (0.61,2.68)	0.509	-0.01 (-0.05,0.03)	0.540	-0.02 (-0.06,0.01)	0.172
1F/2	185	1.33 (0.88,2.02)	0.178	1.37 (0.84,2.24)	0.207	-0.03 (-0.05,0.001)	0.060	-0.02 (-0.04,0.004)	0.108	82	0.74 (0.35,1.55)	0.423	0.67 (0.29,1.52)	0.335	0.03 (-0.01,0.07)	0.184	0.02 (-0.01,0.06)	0.235
2/2	36	**3.14 (1.55,6.38)**	**0.001**	**2.35 (1.01,5.46)**	**0.047**	**-0.06 (-0.11,-0.01)**	**0.020**	-0.01 (-0.05,0.03)	0.515	15	1.96 (0.61,6.27)	0.257	2.31 (0.63,8.40)	0.205	-0.004 (-0.08,0.07)	0.909	-0.005 (-0.07,0.07)	0.895

Model adjusted for age groups, BMI (normal, overweight, obesity), alcohol (g/day), leisure time physical activity (active $\geq$150 min/week), smoking (never, current, past), vitamin D intake. Traditional nomenclature referring to the protein phenotypes: (a) GC1F (rs7041-T/rs4588-C); (b) GC1S (rs7041-G/rs4588-C) and (c) GC2 (rs7041-T/rs4588-A).

Table S11. Associations between GC gene variants and low hip BMD by status menopausal.

	Premenopausal women									Postmenopausal women								
		Categorical hip BMD				Continuous hip BMD					Categorical hip BMD				Continuous hip BMD			
		Crude model		Adjusted model		Crude model		Adjusted model			Crude model		Adjusted model		Crude model		Adjusted model	
	n	OR (95% CI)	*P* value	OR (95% CI)	*P* value	β (95% CI)	*P* value	β (95% CI)	*P* value	n	OR (95% CI)	*P* value	OR (95% CI)	*P* value	β (95% CI)	*P* value	β (95% CI)	*P* value
rs4588																		
CC	305	Ref.		Ref.		Ref.		Ref.		468	Ref.		Ref.		Ref.		Ref.	
CA+AA	200	1.62 (0.97,2.73)	0.066	1.44 (0.80,2.62)	0.228	-0.03 (-0.05,-0.004)	0.022	-0.02 (-0.04,0.01)	0.149	295	1.00 (0.74,1.35)	0.996	1.03 (0.73,1.47)	0.859	-0.007 (-0.03,0.01)	0.489	-0.005 (-0.02,0.01)	0.596
rs7041																		
TT	139	Ref.		Ref.		Ref.		Ref.		216	Ref.		Ref.		Ref.		Ref.	
TG	260	0.62 (0.35,1.09)	0.099	0.56 (0.29,1.07)	0.086	0.02 (-0.01,0.05)	0.142	0.02 (-0.007,0.04)	0.176	355	0.98 (0.69,1.38)	0.895	0.87 (0.62,1.21)	0.410	0.001 (-0.02,0.02)	0.935	0.005 (-0.02,0.02)	0.639
GG	106	0.48 (0.22,1.04)	0.062	0.51 (0.51,1.18)	0.116	**0.04 (0.01,0.07)**	**0.008**	**0.03 (-0.01,0.06)**	**0.017**	193	0.66 (0.44,1.01)	0.054	**0.65 (0.43,0.98)**	**0.040**	0.02 (-0.006,0.05)	0.128	0.01 (-0.009,0.04)	0.246
Haplotype																		
1S/1S	104	Ref.		Ref.		Ref.		Ref.		182	Ref.		Ref.		Ref.		Ref.	
1F/1S	148	1.06 (0.46,2.46)	0.892	0.87 (0.35,2.19)	0.775	-0.01 (-0.05,0.02)	0.390	-0.01 (-0.04,0.02)	0.394	205	1.51 (0.99,2.28)	0.054	0.91 (0.37,2.25)	0.866	-0.02 (-0.05,0.01)	0.117	-0.01 (-0.03,0.02)	0.580
1F/1F	53	1.67 (0.62,4.52)	0.312	2.03 (0.69,5.93)	0.197	**-0.05 (-0.08,-0.003)**	**0.037**	**-0.04 (-0.08,-0.006)**	**0.021**	81	1.29 (0.75,2.23)	0.356	1.75 (0.61,5.03)	0.233	-0.02 (-0.06,0.02)	0.304	-0.02 (-0.05,0.01)	0.194
1S/2	112	1.57 (0.68,3.63)	0.295	1.33 (0.54,3.30)	0.537	**-0.04 (-0.07,-0.005)**	**0.025**	-0.03 (-0.06,0.002)	0.071	148	1.20 (0.76,1.89)	0.439	1.54 (0.64,3.72)	0.325	-0.02 (-0.05,0.01)	0.196	-0.01 (-0.04,0.01)	0.332
1F/2	74	1.64 (0.66,4.09)	0.288	1.34 (0.49,3.68)	0.570	**-0.04 (-0.08,-0.0001)**	**0.049**	-0.03 (-0.06,0.006)	0.106	111	1.37 (0.84,2.24)	0.210	1.54 (0.57,4.14)	0.475	-0.02 (-0.05,0.01)	0.232	-0.01 (-0.04,0.01)	0.367
2/2	12	**9.40 (2.54,34.70)**	**0.001**	**6.88 (1.51,31.4)**	**0.013**	**-0.08 (-0.16,-0.004)**	**0.040**	-0.04 (-0.10,0.03)	0.310	24	2.08 (0.88,4.92)	0.093	**8.46 (1.97,36.30)**	**0.004**	-0.04 (-0.10,0.02)	0.211	0.0004 (-0.05,0.05)	0.986

Model adjusted for age groups, BMI (normal, overweight, obesity), alcohol (g/day), leisure time physical activity (active ≥150 min/week), smoking (never, current, past), vitamin D intake. Traditional nomenclature referring to the protein phenotypes: (a) GC1F (rs7041-T/rs4588-C); (b) GC1S (rs7041-G/rs4588-C) and (c) GC2 (rs7041-T/rs4588-A).

Table S12. Spearman correlation between VDBP levels, different forms of 25(OH)D and BMD by menopausal status.

	Premenopausal Women						Postmenopausal women					
	Hip BMD g/cm^2		Femoral neck g/cm^2		Lumbar spine g/cm^2		Hip BMD g/cm^2		Femoral neck g/cm^2		Lumbar spine g/cm^2	
	r	*P* value	r	*P* value	r	*P* value	r	*P* value	r	*P* value	r	*P* value
VDBP levels (µmol/l)	-0.046	0.300	-0.003	0.944	-0.043	0.333	0.158	<0.001	0.140	<0.001	0.026	0.470
Total 25-hydroxivitamin D (ng/mL)	-0.026	0.264	-0.031	0.483	0.042	0.346	0.083	0.021	0.119	0.0009	0.080	0.025
Free 25-hydroxivitamin D (pg/mL) [a]	-0.007	0.874	-0.012	0.777	0.053	0.211	-0.007	0.861	0.025	0.506	0.044	0.240
Bioavailable 25-hydroxivitamin D (ng/mL) [a]	-0.016	0.712	-0.007	0.871	0.060	0.158	0.009	0.811	0.047	0.203	0.060	0.107
Free 25-hydroxivitamin D-SNP adjusted (pg/mL)	0.029	0.513	0.036	0.427	0.057	0.203	-0.003	0.942	0.010	0.795	0.027	0.464
Bioavailable 25-hydroxivitamin D-SNP adjusted (ng/mL)	0.002	0.701	0.035	0.431	0.059	0.186	0.008	0.838	0.025	0.423	0.039	0.286

Table S13. Spearman correlation between VDBP levels, different forms of 25(OH)D and BMD by sex.

	All Women						All men					
	Hip BMD g/cm^2		Femoral neck g/cm^2		Lumbar spine g/cm^2		Hip BMD g/cm^2		Femoral neck g/cm^2		Lumbar spine g/cm^2	
	r	*P* value	r	*P* value	r	*P* value	r	*P* value	r	*P* value	r	*P* value
VDBP levels (µmol/l)	0.089	0.002	0.096	0.005	0.032	0.248	-0.057	0.179	-0.074	0.079	-0.061	0.151
Total 25-hydroxivitamin D (ng/mL)	0.015	0.588	0.039	0.161	0.040	0.153	0.047	0.269	0.059	0.159	0.053	0.208
Free 25-hydroxivitamin D (pg/mL) [a]	-0.021	0.451	-0.011	0.701	0.016	0.573	-0.078	0.064	-0.100	0.019	-0.088	0.036
Bioavailable 25-hydroxivitamin D (ng/mL) [a]	-0.003	0.909	-0.021	0.450	0.020	0.468	-0.063	0.136	-0.055	0.191	-0.095	0.024
Free 25-hydroxivitamin D-SNP adjusted (pg/mL)	-0.007	0.814	-0.001	0.964	0.009	0.752	-0.033	0.445	-0.054	0.206	-0.069	0.104
Bioavailable 25-hydroxivitamin D-SNP adjusted (ng/mL)	-0.007	0.811	0.005	0.851	0.015	0.605	-0.017	0.684	-0.027	0.535	-0.078	0.067

Table S14. Descriptive statistics by sex and BMD status.

Characteristics	Premenopausal women			Postmenopausal women			Men		
	Normal hip BMD n=444	Low hip BMD n=69	*P* value	Normal hip BMD n=484	Low hip BMD n=292	*P* value	Normal hip BMD n=463	Low hip BMD n=100	*P* value
Total 25-hydroxivitamin D (ng/mL)[a]	20.4 (19.7-21.1)	19.6 (17.9-21.2)	0.103	21.4 (20.8-22.0)	20.5 (19.7-21.0)	0.982	22.1 (20.6-23.6)	22.1 (20.6-23.6)	0.205
Vitamin D-binding protein (µmol/l)[a]	278.5 (271-285.6)	285.3 (267.7-303.0)	0.601	282.4 (274.3-289.6)	258.1 (249.0-267.1)	0.009	264.5 (258.1-270.9)	263.8 (250.0-277.7)	0.757
Free 25-hydroxivitamin D (pg/mL)[a]	5.6 (5.4-5.8)	5.2 (4.6-5.8)	0.062	5.8 (5.6-6.1)	5.8 (5.5-6.1)	0.743	6.3 (6.1-6.6)	6.1 (5.5-6.6)	0.107
Bioavailable 25-hydroxivitamin D (ng/mL)[a]	2.1 (2.0-2.2)	2.0 (1.7-2.2)	0.066	2.2 (2.1-2.3)	2.2 (2.1-2.3)	0.825	2.4 (2.3-2.5)	2.3 (2.1-2.5)	0.060
Free 25-hydroxivitamin D-SNP adjusted (pg/mL)[a]	6.4 (6.0-6.7)	5.7 (4.8-6.5)	0.159	6.5 (6.2-6.8)	6.7 (6.3-7.2)	0.144	7.3 (6.9-7.6)	6.7 (6.0-7.5)	0.052
Bioavailable 25-hydroxivitamin D-SNP adjusted (ng/mL)[a]	2.4 (2.2-2.5)	2.1 (1.7-2.4)	0.121	2.5 (2.4-2.6)	2.5 (2.3-2.6)	0.690	2.8 (2.7-2.9)	2.6 (2.3-2.9)	0.037
	Normal femoral neck BMD n=411	Low femoral neck BMD n=102	*P* value	Normal femoral neck BMD n=331	Low femoral neck BMD n=442	*P* value	Normal femoral neck BMD n=408	Low femoral neck BMD n=151	*P* value
Total 25-hydroxivitamin D (ng/mL)[a]	20.4 (19.7-21.0)	19.6 (18.4-21.0)	0.025	21.8 (21.1-22.5)	20.6 (20.0-21.2)	0.718	21.9 (21.2-22.7)	22.5 (21.4-23.7)	0.578
Vitamin D-binding protein (µmol/l)[a]	278.2 (271.0-285.4)	283.7 (269.9-297.5)	0.528	283.6 (275.1-292.1)	265.2 (258.0-272.4)	0.075	265.2 (258.1,272.3)	262.8 (251.1-274.4)	0.852
Free 25-hydroxivitamin D (pg/mL)[a]	5.6 (5.3-5.8)	5.3 (4.8-5.8)	0.133	5.9 (5.7-6.2)	5.8 (5.5-6.0)	0.877	6.3 (6.0-6.6)	6.4 (5.9-6.8)	0.371
Bioavailable 25-hydroxivitamin D (ng/mL)[a]	2.1 (1.0-2.2)	2.0 (1.8-2.2)	0.154	2.2 (2.1-2.3)	2.2 (2.1-2.3)	0.967	2.4 (2.3-2.5)	2.4 (2.2-2.6)	0.571
Free 25-hydroxivitamin D-SNP adjusted (pg/mL)[a]	6.4 (6.0-6.7)	5.7 (4.9-6.4)	0.143	6.6 (6.2-7.0)	6.5 (6.2-6.9)	0.975	7.3 (6.9-7.6)	7.2 (6.7-7.8)	0.273
Bioavailable 25-hydroxivitamin D-SNP adjusted (ng/mL)[a]	2.4 (2.2-2.5)	2.1 (1.9-2.4)	0.181	2.6 (2.4-2.7)	2.4 (2.3-2.6)	0.587	2.8 (2.6-2.9)	2.8 (2.6-3.0)	0.768
	Normal lumbar spine BMD n=373	Low lumbar spine BMD n=140	*P* value	Normal lumbar spine BMD n=227	Low lumbar spine BMD n=546	*P* value	Normal lumbar spine BMD n=318	Low lumbar spine BMD n=245	*P* value
Total 25-hydroxivitamin D (ng/mL)[a]	20.8 (20.0-21.6)	19.5 (18.3-20.8)	0.037	21.3 (20.5-22.2)	21.0 (20.5-21.6)	0.786	21.3 (20.5-22.2)	22.6 (21.7-23.6)	0.157
Vitamin D-binding protein (µmol/l)[a]	278.6 (270.9-286.3)	281.3 (269.0-293.6)	0.615	272.8 (261.8-283.8)	271.7 (264.9-278.6)	0.625	267.0 (259.4-274.6)	260.5 8251.8-269.2)	0.142
Free 25-hydroxivitamin D (pg/mL)[a]	5.6 (5.3-5.9)	5.3 (4.9-5.7)	0.145	5.9 (5.6-6.2)	5.8 (5.6-6.0)	0.780	5.9 (5.6-6.2)	6.5 (6.2-6.9)	0.021
Bioavailable 25-hydroxivitamin D (ng/mL)[a]	2.1 (2.0-2.2)	2.0 (1.8-2.2)	0.125	2.2 (2.1-2.4)	2.2 (2.1-2.3)	0.687	2.3 (2.2-2.4)	2.5 (2.4-2.7)	0.021
Free 25-hydroxivitamin D-SNP adjusted (pg/mL)[a]	6.4 (6.0-6.8)	5.9 (5.2-6.5)	0.258	6.6 (6.1-7.1)	6.6 (6.3-6.9)	0.861	7.0 (6.6-7.4)	7.5 (7.0-8.0)	0.267

Bioavailable 25-hydroxivitamin D-SNP adjusted (ng/mL) [a]	2.4 (2.2-2.5)	2.2 (2.0-2.4)	0.283	2.5 (2.3-2.7)	2.5 (2.4-2.6)	0.854	2.7 (2.5-2.9)	2.9 (2.7-3.1)	0.306

[a] Adjusted median (95% CI). Median adjusted by age groups, BMI (normal, overweight, obesity), leisure time physical activity (active >150 min/week), vitamin D intake (quartiles) and smoking (never, smoking, former smoker). Low BMD was defined as a T-score less than -1 at the femoral neck, lumbar spine and hip, according to WHO criteria.

Table S15. Association between VDBP categories and low hip BMD in all participants.

	All participants n=1,853 VDBP tertiles					All women n=1,289 VDBP tertiles				
Model	Low (106.8-246.3) OR (95% CI)	Medium (246.4-299.5) OR (95% CI)	*P* value	High (299.52,449.7) OR (95% CI)	*P* value	Low (106.8-250.5)	Medium (250.9-304.1)		High (304.2-449.7)	
Crude	Ref.	**0.71 (0.55,0.92)**	**0.010**	**0.71 (0.55,0.92)**	**0.010**	Ref.	**0.69 (0.52,0.92)**	**0.012**	**0.53 (0.39,0.72)**	**<0.001**
Adjusted[1]	Ref.	0.84 (0.63,1.13)	0.255	0.78 (0.58,1.01)	0.103	Ref.	0.79 (0.56,1.11)	0.181	**0.67 (0.46,0.95)**	**0.026**
Adjusted[2]	Ref.	0.86 (0.64,1.16)	0.332	0.80 (0.59,1.08)	0.137	Ref.	0.80 (0.57,1.13)	0.217	**0.67 (0.47,0.96)**	**0.029**

Model 1 adjusted for age groups, sex, BMI (normal, overweight, obesity), alcohol (g/day), leisure time physical activity (active >30 min/day) and smoking (never, smoking, former smoker). Model 2 adjusted for age groups, sex, BMI (normal, overweight, obesity), alcohol (g/day), leisure time physical activity (active >30 min/day), smoking (never, current, past), vitamin D intake and 25-hydroxivitamin D levels. a Low, medium and high VDBP category levels were defined by tertiles. *Minimum -maximum value of each category.

Table S16. Association between VDBP levels and BMD by menopausal status.

Model	Premenopausal, n=513						Postmenopausal, n=776					
	Hip BMD (g/cm^2) β (95% CI)	P value	Femoral neck (g/cm^2) β (95% CI)	P value	Spine lumbar (g/cm^2) β (95% CI)	P value	Hip BMD (g/cm^2) β (95% CI)	P value	Femoral neck (g/cm^2) β (95% CI)	P value	Spine lumbar (g/cm^2) β (95% CI)	P value
Crude	-0.02 (-0.07,0.03)	0.479	0.009 (-0.04,0.06)	0.705	-0.01 (-0.06,0.04)	0.579	**0.07 (0.03,0.11)**	**<0.001**	**0.06 (0.03,0.10)**	**0.001**	0.03 (-0.02,0.07)	0.213
Adjusted[1]	-0.009 (-0.05,0.04)	0.699	0.006 (-0.04,0.05)	0.783	-0.02 (-0.07,0.03)	0.468	0.02 (-0.01,0.06)	0.165	0.02 (-0.01,0.05)	0.235	0.01 (-0.03,0.05)	0.650
Adjusted[2]	-0.01 (-0.05,0.04)	0.733	0.009 (-0.03,0.05)	0.694	-0.01 (-0.06.0.04)	0.615	0.02 (-0.01,0.06)	0.186	0.02 (-0.01,0.05)	0.284	0.008 (-0.04,0.05)	0.721

* VDBP levels were logarithmically transformed to the base 10. Model 1 adjusted for age groups, BMI (normal, overweight, obesity), alcohol (g/day), leisure time physical activity (active ≥30 min/day) and smoking (never, smoking, former smoker). Model 2 adjusted for age groups, BMI (normal, overweight, obesity), alcohol (g/day), leisure time physical activity (active ≥30 min/day), smoking (never, current, past), vitamin D intake and 25-hydroxivitamin D levels.

Table S17. Association between VDBP levels and BMD by VitD status.

	Normal Vitamin D, n=1,077						Deficiency Vitamin D, n=775					
Model	Hip BMD (g/cm^2) β (95% CI)	P value	Femoral neck (g/cm^2) β (95% CI)	P value	Spine lumbar (g/cm^2) β (95% CI)	P value	Hip BMD (g/cm^2) β (95% CI)	P value	Femoral neck (g/cm^2) β (95% CI)	P value	Spine lumbar (g/cm^2) β (95% CI)	P value
Crude	0.03 (-0.01,0.07)	0.090	**0.05 (0.01,0.09)**	**0.008**	**0.04 (0.001,0.09)**	**0.047**	0.04 (-0.003,0.09)	0.069	0.05 (-0.002,0.09)	0.059	0.01 (-0.04,0.06)	0.797
Adjusted[1]	0.02 (-0.01,0.05)	0.281	0.03 (-0.004,0.06)	0.095	0.03 (-0.09,0.07)	0.123	0.03 (-0.002,0.07)	0.068	0.03 (-0.006,0.07)	0.102	0.007 (-0.04,0.05)	0.775
Adjusted[2]	0.02 (-0.02,0.05)	0.302	0.03 (-0.005,0.06)	0.100	0.03 (-0.01,0.07)	0.128	0.04 (-0.001,0.07)	0.063	0.03 (-0.005,0.07)	0.090	0.009 (-0.04,0.05)	0.699

* VDBP levels were logarithmically transformed to the base 10. Model 1 adjusted for age groups, sex, BMI (normal, overweight, obesity), alcohol (g/day), leisure time physical activity (active ≥30 min/day) and smoking (never, smoking, former smoker). Model 2 adjusted for age groups, sex, BMI (normal, overweight, obesity), alcohol (g/day), leisure time physical activity (active ≥30 min/day), smoking (never, current, past) and vitamin D intake.

Table S18. Association between VitD levels and BMD in all population.

	All participants, n=1,853					
	Total 25 hydroxivitamin D					
	Hip BMD (g/cm^2)		Femoral neck (g/cm^2)		Spine lumbar (g/cm^2)	
Model	β (95% CI)	P value	β (95% CI)	P value	β (95% CI)	P value
Crude	0.02 (-0.0008,0.05)	0.059	**0.03 (0.003,0.05)**	**0.030**	0.03 (-0.001,0.05)	0.055
Adjusted[1]	0.0007 (-0.0002,0.002)	0.109	**0.02 (0.003,0.04)**	**0.020**	0.02 (-0.006,0.04)	0.152
Adjusted[2]	0.02 (-0.002,0.04)	0.073	**0.02 (0.002,0.04)**	**0.027**	0.02 (-0.007,0.04)	0.178
	Free 25 hydroxivitamin D					
Crude	0.007 (-0.009,0.02)	0.404	0.006 (-0.01,0.02)	0.455	0.008 (-0.01,0.03)	0.393
Adjusted[1]	0.002 (-0.01,0.02)	0.717	0.004 (-0.01,0.02)	0.532	0.004 (-0.01,0.02)	0.629
Adjusted[2]	0.002 (-0.01,0.02)	0.711	0.004 (-0.008,0.02)	0.519	0.004 (-0.01,0.02)	0.618
	Bioavailable 25 hydroxivitamin D					
Crude	0.01 (-0.01,0.03)	0.347	0.02 (-0.004,0.04)	0.124	0.007 (-0.02,0.03)	0.535
Adjusted[1]	0.005 (-0.01,0.02)	0.522	0.01 (-0.004,0.03)	0.156	0.002 (-0.02,0.02)	0.771
Adjusted[2]	0.005 (-0.01,0.02)	0.507	0.01 (-0.004,0.03)	0.152	0.003 (-0.02,0.02)	0.801

*Vitamin D levels (total, free and bioavailable 25 hydroxyvitamin D) were logarithmically transformed to the base 10. Model 1 adjusted for age groups, sex, BMI (normal, overweight, obesity), alcohol (g/day), leisure time physical activity (active ≥30 min/day) and smoking (never, smoking, former smoker). Model 2 adjusted for age groups, sex, BMI (normal, overweight, obesity), alcohol (g/day), leisure time physical activity (active ≥30 min/day), smoking (never, current, past) and vitamin D intake.

Table S19. Association between VitD levels and BMD by sex.

Model	All women, n=1289						All men, n=564					
	Total 25 hydroxivitamin D											
	Hip BMD (g/cm^2) β (95% CI)	P value	Femoral neck (g/cm^2) β (95% CI)	P value	Spine lumbar (g/cm^2) β (95% CI)	P value	Hip BMD (g/cm^2) β (95% CI)	P value	Femoral neck (g/cm^2) β (95% CI)	P value	Spine lumbar (g/cm^2) β (95% CI)	P value
Crude	0.01 (-0.02,0.04)	0.415	0.02 (-0.005,0.05)	0.111	0.02 (-0.01,0.05)	0.217	**-0.02 (-0.06,0.02)**	**0.438**	**-0.02 (-0.07,0.02)**	**0.312**	-0.01 (-0.06,0.03)	0.549
Adjusted[1]	0.0004 (-0.001,0.001)	0.421	**0.02 (0.001,0.04)**	**0.036**	0.02 (-0.002,0.05)	0.070	0.001 (-0.003,0.003)	0.120	0.02 (-0.01,0.06)	0.221	0.009 (-0.04,0.05)	0.695
Adjusted[2]	0.013 (-0.008,0.03)	0.229	**0.02 (0.0003,0.04)**	**0.047**	0.02 (-0.003,0.05)	0.087	0.02 (-0.01,0.06)	0.202	0.02 (-0.02,0.06)	0.287	0.006 (-0.04,0.05)	0.778
	Free 25 hydroxivitamin D											
Crude	-0.005 (-0.02,0.01)	0.594	-0.004 (-0.02,0.01)	0.644	0.003 (-0.02,0.02)	0.764	-0.008 (-0.04,0.02)	0.573	-0.008 (-0.04,0.02)	0.583	-0.02 (-0.05,0.02)	0.324
Adjusted[1]	0.001 (-0.01,0.02)	0.857	0.003 (-0.01,0.02)	0.642	0.01 (-0.005,0.03)	0.150	0.006 (-0.02,0.03)	0.623	0.008 (-0.02,0.03)	0.569	0.01 (-0.005,0.03)	0.150
Adjusted[2]	0.001 (-0.01,0.02)	0.864	0.003 (-0.01,0.02)	0.640	0.01 (-0.01,0.03)	0.158	0.006 (-0.02,0.03)	0.620	0.007 (-0.02,0.03)	0.577	0.01 (-0.005,0.03)	0.158
	Bioavailable 25 hydroxivitamin D											
Crude	-0.008 (-0.03,0.01)	0.510	-0.002 (-0.02,0.02)	0.852	-0.0008 (-0.03,0.02)	0.953	-0.008 (-0.03,0.01)	0.510	-0.002 (-0.02,0.02)	0.852	-0.0008 (-0.03,0.02)	0.953
Adjusted[1]	0.006 (-0.01,0.02)	0.542	0.01 (-0.007,0.03)	0.231	0.01 (-0.009,0.04)	0.229	0.006 (-0.01,0.02)	0.542	0.01 (-0.007,0.03)	0.231	0.01 (-0.009,0.04)	0.229
Adjusted[2]	0.006 (-0.01,0.02)	0.536	0.01 (-0.007,0.03)	0.246	0.01 (-0.009,0.04)	0.250	0.005 (-0.01,0.02)	0.536	0.01 (-0.007,0.03)	0.246	0.01 (-0.01,0.04)	0.250

* Vitamin D levels (total, free and bioavailable 25 hydroxyvitamin D) were logarithmically transformed to the base 10. Model 1 adjusted for age groups, BMI (normal, overweight, obesity), alcohol (g/day), leisure time physical activity (active ≥30 min/day) and smoking (never, smoking, former smoker). Model 2 adjusted for age groups, BMI (normal, overweight, obesity), alcohol (g/day), leisure time physical activity (active ≥30 min/day), smoking (never, current, past) and vitamin D intake.

Table S20. Association between VitD levels and BMD by menopausal status.

Model	Premenopausal, n=513						Postmenopausal, n=776					
	Hip BMD (g/cm^2) β (95% CI)	P value	Femoral neck (g/cm^2) β (95% CI)	P value	Spine lumbar (g/cm^2) β (95% CI)	P value	Hip BMD (g/cm^2) β (95% CI)	P value	Femoral neck (g/cm^2) β (95% CI)	P value	Spine lumbar (g/cm^2) β (95% CI)	P value
	Total 25 hydroxivitamin D											
Crude	-0.01 (-0.05,0.03)	0.544	0.001 (-0.03,0.04)	0.950	0.02 (-0.02,0.06)	0.245	**0.05 (0.01,0.08)**	**0.008**	**0.06 (0.03,0.09)**	**<0.001**	0.04 (-0.003.0.08)	0.037
Adjusted[1]	0.001 (-0.001,0.002)	0.497	0.02 (-0.01,0.06)	0.169	0.03 (-0.01,0.07)	0.116	0.0005 (-0.0009,0.002)	0.506	0.03 (-0.003,0.05)	0.075	0.02 (-0.01,0.06)	0.234
Adjusted[2]	0.01 (-0.02,0.05)	0.380	0.02 (-0.01,0.05)	0.182	0.03 (-0.009,0.06)	0.134	0.02 (-0.01,0.04)	0.308	0.02 (-0.004,0.05)	0.100	0.02 (-0.02,0.06)	0.262
	Free 25 hydroxivitamin D											
Crude	0.003 (-0.02,0.03)	0.836	0.004 (-0.02,0.03)	0.748	0.01 (-0.01,0.04)	0.387	0.003 (-0.02,0.03)	0.800	0.007 (-0.02,0.03)	0.558	0.02 (-0.01,0.04)	0.217
Adjusted[1]	0.002 (-0.02,0.02)	0.843	0.005 (-0.02,0.03)	0.666	0.01 (-0.01,0.04)	0.256	0.001 (-0.02,0.02)	0.895	0.003 (-0.02,0.02)	0.746	0.01 (-0.01,0.04)	0.256
Adjusted[2]	0.002 (-0.02,0.02)	0.826	0.005 (-0.02,0.03)	0.643	0.01 (-0.01,0.04)	0.275	0.001 (-0.02,0.02)	0.923	0.003 (-0.02,0.02)	0.763	0.01 (-0.01,0.04)	0.275
	Bioavailable 25 hydroxivitamin D											
Crude	-0.001 (-0.03,0.03)	0.920	0.01 (-0.02,0.04)	0.441	0.008 (-0.03,0.04)	0.649	-0.001 (-0.03,0.03)	0.920	0.01 (-0.02,0.04)	0.441	0.008 (-0.03,0.04)	0.649
Adjusted[1]	0.001 (-0.02,0.03)	0.955	0.009 (-0.01,0.03)	0.460	0.01 (-0.02,0.04)	0.533	0.001 (-0.02,0.03)	0.955	0.009 (-0.01,0.03)	0.460	0.01 (-0.02,0.04)	0.533
Adjusted[2]	0.001 (-0.02,0.03)	0.950	0.008 (-0.02,0.03)	0.499	0.01 (-0.02,0.04)	0.562	0.001 (-0.02,0.03)	0.950	0.008 (-0.02,0.03)	0.499	0.01 (-0.02,0.04)	0.562

Vitamin D levels (total, free and bioavailable 25 hydroxyvitamin D) were logarithmically transformed to the base 10. Model 1 adjusted for age groups, BMI (normal, overweight, obesity), alcohol (g/day), leisure time physical activity (active ≥30 min/day) and smoking (never, smoking, former smoker). Model 2 adjusted for age groups, BMI (normal, overweight, obesity), alcohol (g/day), leisure time physical activity (active ≥30 min/day), smoking (never, current, past) and vitamin D intake.
